# Upscaling the urea method synthesis of CoAl layered double hydroxides

**DOI:** 10.3762/bjnano.14.76

**Published:** 2023-09-11

**Authors:** Camilo Jaramillo-Hernández, Víctor Oestreicher, Martín Mizrahi, Gonzalo Abellán

**Affiliations:** 1 Instituto de Ciencia Molecular (ICMol). Universidad de Valencia, Catedrático José Beltrán 2, Paterna, Valencia, 46980, Spain; 2 Instituto de Investigaciones Fisicoquímicas Teóricas y Aplicadas (INIFTA), Departamento de Química, Facultad de Ciencias Exactas. Universidad Nacional de La Plata, CCT La Plata- CONICET. Diagonal 113 y 64, 1900, La Plata, Argentina; 3 Facultad de Ingeniería, Universidad Nacional de La Plata. Calle 1 esq. 47, 1900, La Plata, Argentina

**Keywords:** Co-based hydroxides, layered double hydroxide, layered materials, scale-up process, synthesis, two-dimensional materials

## Abstract

Research on two-dimensional materials is one of the most relevant fields in materials science. Layered double hydroxides (LDHs), a versatile class of anionic clays, exhibit great potential in photocatalysis, energy storage and conversion, and environmental applications. However, its implementation in real-life devices requires the development of efficient and reproducible large-scale synthesis processes. Unfortunately, reliable methods that allow for the production of large quantities of two-dimensional LDHs with well-defined morphologies and high crystallinity are very scarce. In this work, we carry out a scale-up of the urea-based CoAl-LDH synthesis method. We thoroughly study the effects of the mass scale-up (25-fold: up to 375 mM) and the volumetric scale-up (20-fold: up to 2 L). For this, we use a combination of several structural (XRD, TGA, and N_2_ and CO_2_ isotherms), microscopic (SEM, TEM, and AFM), magnetic (SQUID), and spectroscopic techniques (ATR-FTIR, UV–vis, XPS, ICP-MS, and XANES-EXAFS). In the case of the volumetric scale-up, a reduction of 45% in the lateral dimensions of the crystals (from 3.7 to 2.0 µm) is observed as the reaction volume increases. This fact is related to modified heating processes affecting the alkalinization rates and, concomitantly, the precipitation, even under recrystallization at high temperatures. In contrast, for the tenfold mass scale-up, similar morphological features were observed and assigned to changes in nucleation and growth. However, at higher concentrations, simonkolleite-like Co-based layered hydroxide impurities are formed, indicating a phase competition during the precipitation related to the thermodynamic stability of the growing phases. Overall, this work demonstrates that it is possible to upscale the synthesis of high-quality hexagonal CoAl-LDH in a reproducible manner. It highlights the most critical synthesis aspects that must be controlled and provides various fingerprints to trace the quality of these materials. These results will contribute to bringing the use of these 2D layered materials closer to reality in different applications of interest.

## Introduction

Since the discovery of graphene [1], research on two-dimensional (2D) materials has become one of the most relevant topics in physics, chemistry, and (nano)materials science [2–4]. These materials play a key role both from a fundamental point of view and regarding potential applications in electronic devices, drug delivery, and energy storage and conversion, to name a few [5–8].

Layered materials range from monoelementals (i.e., graphene, silicene, germanene, or pnictogens (P, As, Sb and Bi)) to multielementals (e.g., boron nitrate, metal dichalcogenides, MXenes, layered metal/covalent organic frameworks, or layered hydroxides/oxides) [9–11]. These systems exhibit an enormous variability in their physicochemical properties, which are defined by their layer-to-layer interactions and chemical composition.

One of the most interesting families is that of layered double hydroxides (LDHs), which are characterized by having a positive charge, hence the name “anionic clays”. This family exhibits hydrotalcite-like structures consisting of infinite positively charged layers containing M^II^ and M^III^ octahedral cations connected by μ_3_-OH bridges that interact electrostatically with interlayer anions. Typically, LDHs can be represented by the chemical formula 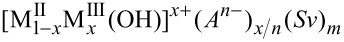, where M represents cations (e.g., Mg, Zn, Co, Ni, Cu, Al, Fe, or Cr) and *x* the metallic ratio (typically, 0.20 < *x* < 0.33). *A^n^*^−^ symbolizes a constituent ranging from (in)organic anions to macromolecules, and *Sv* stands for solvent molecules. This general composition leads to a plethora of highly tunable systems [12–16] with relevance in environmental applications [17], photocatalysis [18], energy storage and conversion [19–21], quantum materials [22–23], and others [24]. This wide range of potential applications makes the development of reliable scaling processes crucial.

Usually, LDHs are obtained by different synthesis procedures such as co-precipitation [25], hydrothermal synthesis [13], sol–gel methods [26], mechanochemistry [27], or the epoxide route [28], to name a few [29]. Among them, hydrothermal methods based on ammonium-releasing reagents (ARRs), commonly urea or hexamethylenetetramine, are especially interesting since they allow one to obtain large and highly crystalline particles [30–32]. The ARR decomposes at temperatures above 70 °C, which leads to the alkalinization of a solution containing cation reagents, eventually triggering the precipitation of LDHs [13,33–35]. The experimental conditions (concentration, solvent mixture, and temperature) will define the alkalinization rate, which (mainly) controls the nucleation and growth processes, and therefore particle size, morphology, and crystallinity [33,36–37].

Attempts to upscale the production of LDHs included incrementing the concentration of the reactants [38–39], the use of large-scale reactors [40–41], byway co-precipitation, and mechanochemical approaches [27]. Although these methods can produce materials on a large scale, they are very limited in providing materials with controlled morphology, size, or crystallinity [42]. This issue can be partially solved using continuous flow techniques [43–44]. Yet, reliable scaling methods that allow for the production of large quantities of two-dimensional LDHs with well-defined morphologies and high crystallinity are very scarce.

Herein, we thoroughly study the scale-up of CoAl-LDH synthesis by a urea alkalization method. We explore both volumetric (increment in reactor size) and mass (increment in the reagent concentration) scale-up processes. In the mass scale-up process, the increment in the concentration (25-fold that of the reference condition) triggers the appereance of simonkolleite-like Co-based impurities due to phase competition during the precipitation process (thermodynamic aspects). In the volumetric approach, pure CoAl-based LDHs are obtained, size and shape (edge sharpness) of which highly depend on the heating procedure, even after 48 h of recrystallization (kinetic aspects). Our results suggest that either an up to tenfold mass scale-up or a 20-fold volumetric scale-up can provide pure CoAl-based LDH materials exhibiting comparable morphology and crystallinity.

Interestingly, while in the case of the volumetric scale-up, the kinetic issues could be solved by a better control over the heating process, the thermodynamic aspects (phase competition) [30,45] exclusively depend on the nature of the involved cations.

## Results and Discussion

**Scheme 1:**
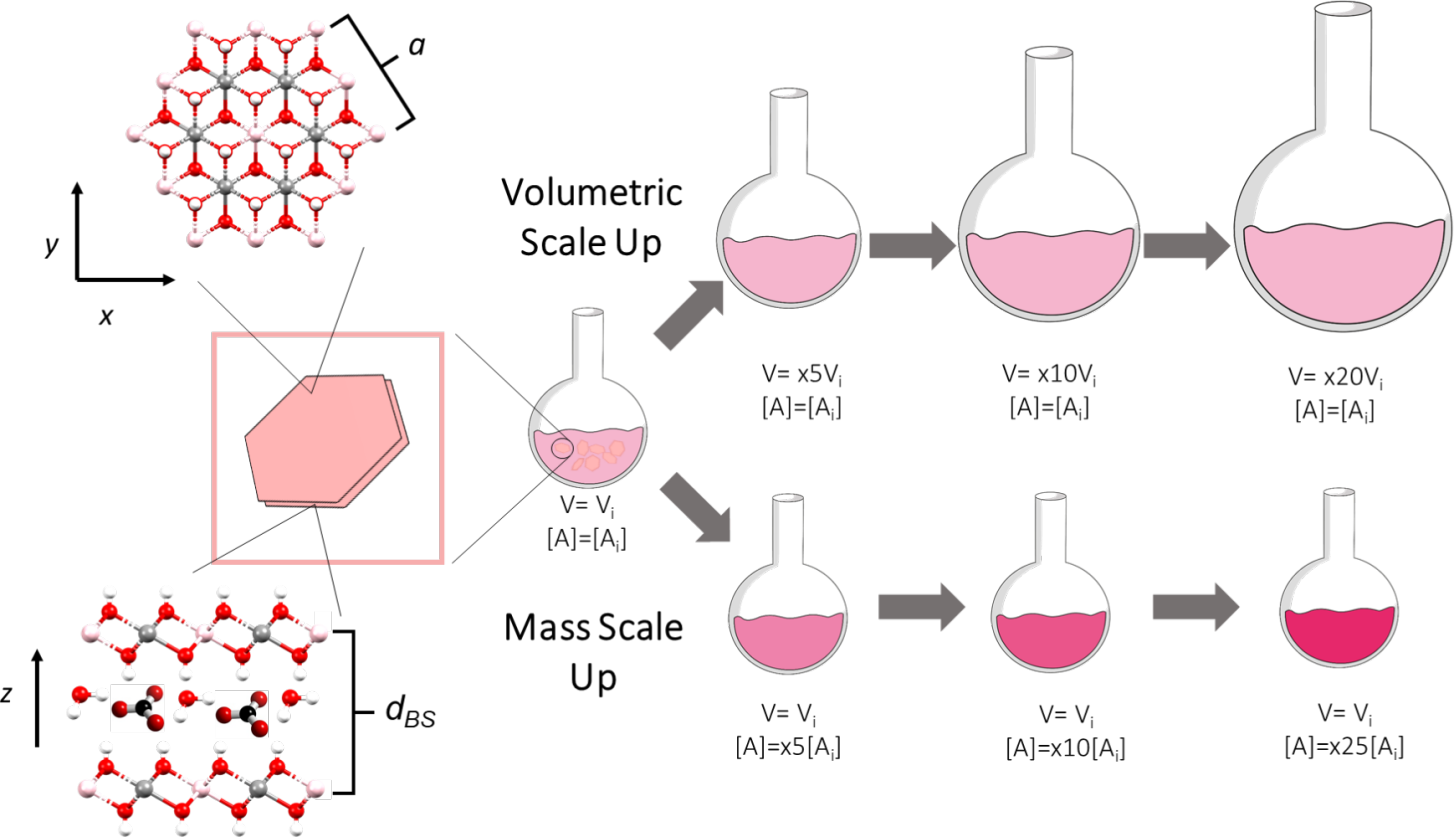
Right: Schematic representation of volumetric and mass scale-up approaches. Left: LDH structure highlighting both crystal facets, *xy* (in-plane) and *z* (out-of-plane) views and the crystallographic parameters basal space distance (*d*_BS_) and parameter *a*.

In order to analyse the effect of the scale-up on the obtained LDH materials, we have selected as reference the experimental conditions for the synthesis of CoAl-LDHs reported by Liu et al. [13], which currently arises as one of the most cited papers describing the synthesis of LDH phases. The aforementioned experimental conditions have been labeled as “x1”. The experiments featuring an increment in the initial volume (i.e., volumetric scale-up) or initial reagent concentration (i.e., mass scale-up) will be designated as x*Y*V and x*Y*M, respectively. Here, *Y* is the factor of the scale-up. In the case of the mass scale-up, a 100 mL two-necked round bottom flask was employed. For the volumetric scale-up, different two-neck round bottom flasks ranging from 500 to 2000 mL were used. In all experiments, the same hotplate stirrer RET Basic (IKA, Germany) was used to keep the temperature at 97 °C. The whole synthesis process (heating, precipitation and cooldown) was carried out under stirring (750 rpm). Scheme 1 depicts the experimental approach of this work, highlighting key structural parameters of the CoAl-based LDH structure.

**Figure 1:**
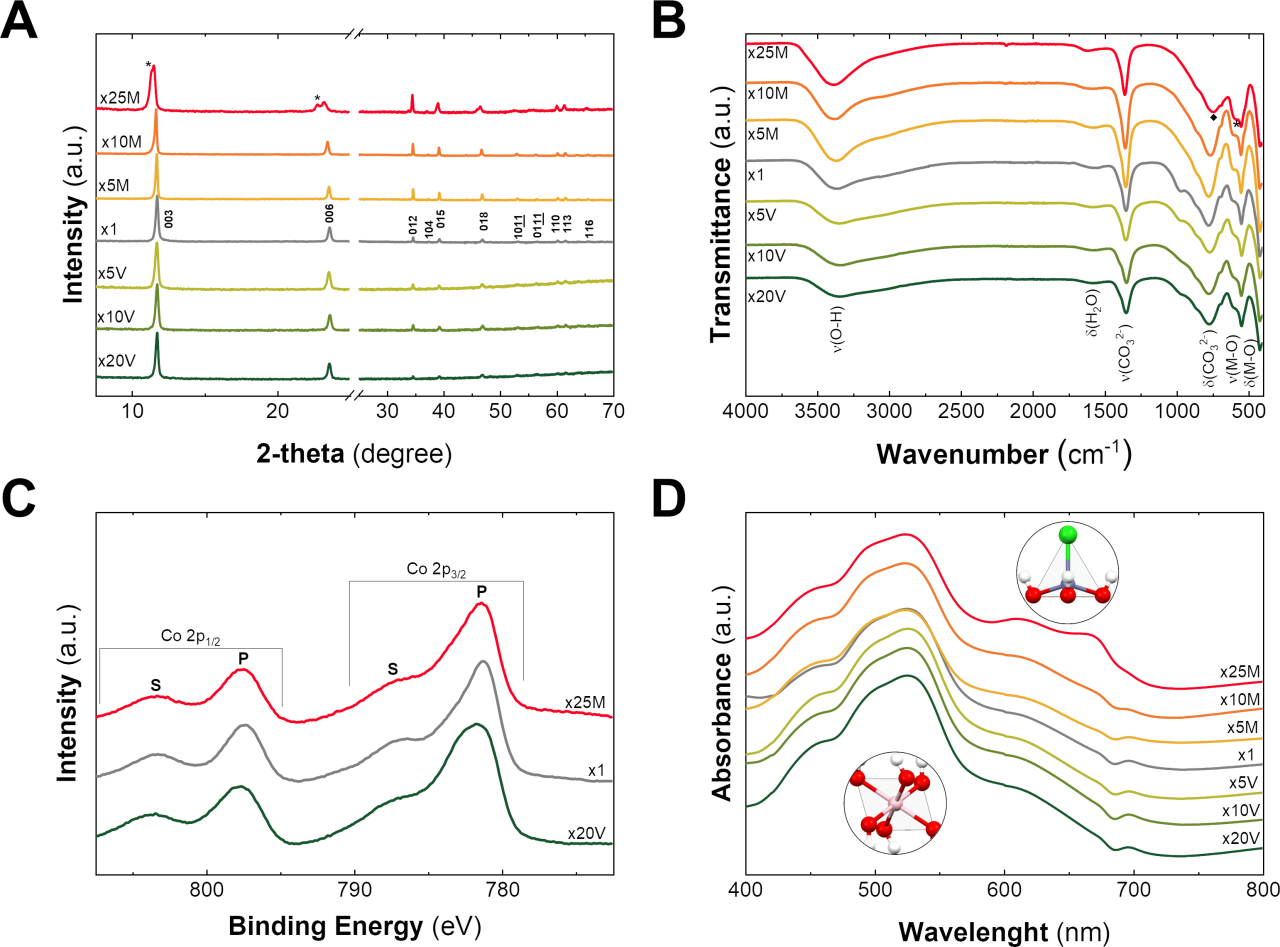
(A) PXRD patterns exhibit the layered nature of the obtained samples. Indexation according to [13]. (B) ATR-FTIR spectra. (C) High-resolution XPS spectra for Co 2p (2p_3/2_ and 2p_1/2_) in the range of 810–770 eV. (D) UV–vis spectra pointing out the marked differences between the octahedral Co^II^(Oh) and tetrahedral environments Co^II^(Td) of cobalt for the scale-up samples.

Figure 1A depicts the PXRD patterns for the obtained pale pink solid samples. The pattern of reference sample x1 exhibits the typical reflections expected for a CoAl-LDH structure. Specifically, the two main signals located at 11.72° and 23.62°, indexed as (003) and (006), are assigned to the interlayer reflections, revealing a basal space distance (*d*_BS_) of 7.56 Å. This value is in perfect agreement with a CoAl LDH phase containing carbonate as interlayer anion [13]. Furthermore, from the signal at around 60°, corresponding to the (110) planes, the parameter *a* (related to the M–O distance) can be estimated to a value of 3.07 Å, which is in agreement with a CoAl-based LDH exhibiting a Co/Al ratio of 2:1 [13,15]. The scale-up samples depict PXRD patterns similar to that of reference x1, suggesting the formation of analogous CoAl LDH phases. However, in the case of sample x25M, the existence of a second set of interlayer reflections (denoted with asterisks in Figure 1A), corresponding to a layered structure with *d*_BS_ = 7.8 Å, suggests the presence of an impurity. Also, the (003) reflection of x10M exhibits an asymmetry in comparison to samples x1 and x10V (see Figure S1 and Figure S2, Supporting Information File 1). Table S1 summarizes the values of *d*_BS_ and parameter *a* for all samples.

Attenuated total reflectance Fourier-transform infrared spectroscopy (ATR-FTIR) provides valuable information about the nature of the layered hydroxide structure and the intercalated anions (Figure 1B). In the case of the reference x1, the spectrum displays a broad signal at ca. 3400 cm^−1^, which corresponds to the OH stretching vibrations typically attributed to interlayer water molecules, as confirmed by the extra signal at 1600 cm^−1^ (water bending mode). The presence of carbonate as interlayer anion is confirmed by the vibration bands centered at 1350 and 775 cm^−1^. Finally, peaks below 750 cm^−1^ are related to the Co/Al–O vibrational bands [13,46–47]. Overall, a CoAl-LDH containing carbonate as interlayer anion is observed. Interestingly, in the case of sample x25M, a shift in the carbonate bending signal (from 775 to 740 cm^−1^, denoted with a diamond) and the appearance of a shoulder at 581 cm^−1^ (denoted with an asterisk) indicate the presence of the impurity already observed by PXRD (see also Supporting Information File 1, Figure S3) [48].

Aiming to determine the identity of the impurity observed in sample x25M, X-ray photoelectron spectroscopy (XPS) was conducted (Figure 1C). In the case of reference x1, the observed main peaks at 781.23 eV (Co 2_3/2_) and 797.36 eV (Co 2_1/2_), as well as their satellites at 783.13 and 798.83 eV, confirm the occurrence of Co^II^ [15,49]. The XPS spectra of the samples x20V and x25M are indistinguishable from that of reference x1, suggesting the lack of Co^III^ in the impurity of sample x25M. Supporting Information File 1, Table S2 compiles further information related to the XPS signals.

Finally, UV–vis spectroscopy has demonstrated to be a powerful technique for layered hydroxide characterization, especially in the case of earth-abundant 3d cations where this technique can provide information about coordination environments and oxidation states [50]. The spectrum of reference x1 depicts a main signal at 525 nm containing high-left and low-right shoulders around 492 and 450 nm. The shape and the position of these d–d electronic transition bands are assigned to the ^4^T_1g_→^4^T_1g_(P) and ^4^T_1g_→^4^A_2g_(F) transitions in octahedral divalent cobalt cations (Co^II^(Oh)) [31,51–52]. However, the sample x25M contains an extra band with a double peak around 650 nm resembling that of Co-based simonkolleite-like structures, also known as α-Co LH [31]. Indeed, this signal can be ascribed to the ^4^A_2_(F)→^4^T_1_(P) transition, corresponding to Co^II^(Td), where the exact position depends on the nature of the coordinated anions [53]. Sample x10M also exhibits this extra band, but less intense and only noticeable when the values of absorbance are normalized (Supporting Information File 1, Figure S4). Thus, considering the peaks at 610 and 665 nm, the impurity could be associated to a simonkolleite-like α-Co^II^ LH structure (see control experiments and further characterization in Figure S5 and Figure S6, Supporting Information File 1). Regarding sample x10M, the asymmetry of the (003) reflection observed in PXRD can be an indicator of the presence of an impurity. Furthermore, we have characterized these samples by conventional SQUID magnetic measurements. Despite the acute differences in the magnetic behavior of Co-based LDH and simonkolleite-like α-LH [46,54–55], the impurities do not lead to significant changes beyond slight variations in the DC magnetic susceptibility and the out-of-phase contribution of the dynamic susceptibility (Figure S7 and Figure S8, Supporting Information File 1).

The appearance of α-Co LH with increasing reagent concentration (sample x25M) indicates a typical precipitation competition scenario, where the initial conditions can modify the relative thermodynamic stability of the growing phases. Indeed, the occurrence of α-Co LH as an impurity in the early precipitation stages has been already reported in the case of the synthesis of β-Co(OH)_2_ [30] and CoAl-based LDHs [45]. Since this phase competition is ruled by thermodynamic aspects, its occurrence will depend on the chemical identity of the involved cations [30,45,56–58]. This has been observed in different large-scale approaches, where different phases besides hydroxides have been observed at high concentrations [59–60].

**Figure 2:**
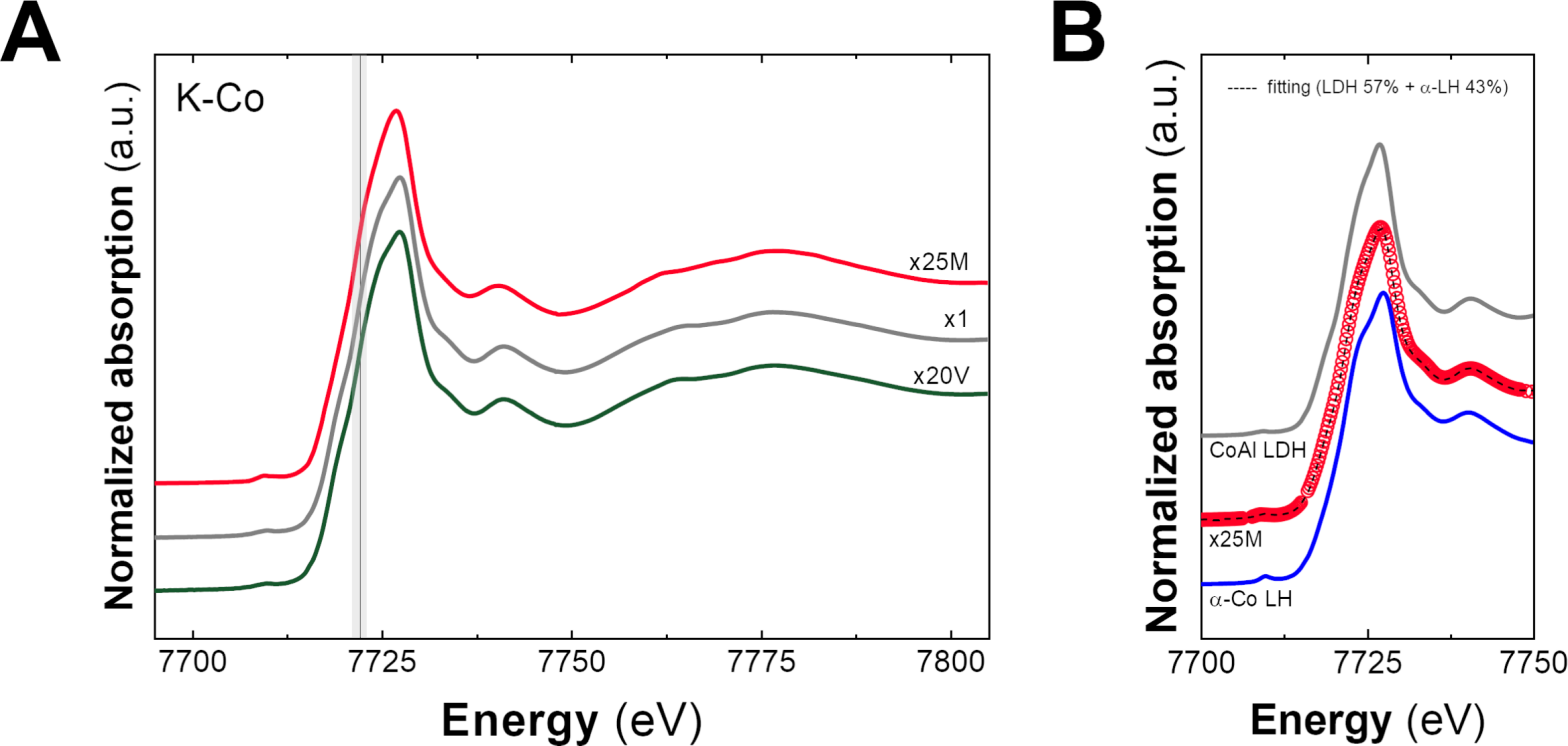
(A) Normalized XANES spectra at the Co K edge for the obtained samples. The grey line depicts the position of the absorption edge characteristic to Co^II^. (B) Linear fit combination by employing CoAl-LDH and α-Co LH as references, suggesting fractions of 57% of LDH and 43% of the impurity.

In order to provide a comprehensive understanding of the electronic and structural features resulting from the scale-up process and aiming to quantify the amount of this Co-based α-LH impurity, X-ray absorption spectroscopy (XAS) measurements were performed at the CLÆSS BL22 beamline at the ALBA synchrotron. Figure 2A depicts the X-ray absorption near-edge structure (XANES) spectra for the Co K edge. In all samples, the presence of Co^II^ is confirmed regardless of the synthesis conditions [31,50,61]. Nevertheless, sample x25M shows differences in the intensity of the white line and in the resonances behind the absorption edge, compared to the other samples studied. At first glance, this would indicate that, although all Co atoms are in the same oxidation state (2+), they would be found in different environments, as suggested by PXRD and confirmed by UV–vis spectroscopy. This means the presence of the α-Co^II^ LH impurity. To quantify its fraction, the spectrum of sample x25M was reproduced by using a linear combination of CoAl-LDH and α-Co^II^ LH reference spectra. Figure 2B depicts the result of the fits, where excellent agreement is achieved using 57% of LDH and 43% of α-Co^II^ LH. This analysis is an excellent example to show that XANES is a useful technique for LH structure quantification. A detailed description of the structural features of the scale-up samples determined by extended X-ray absorption fine structure (EXAFS) measurements, the used model, and the corresponding fits can be found in Supporting Information File 1 (Figure S9 and Table S3).

**Figure 3:**
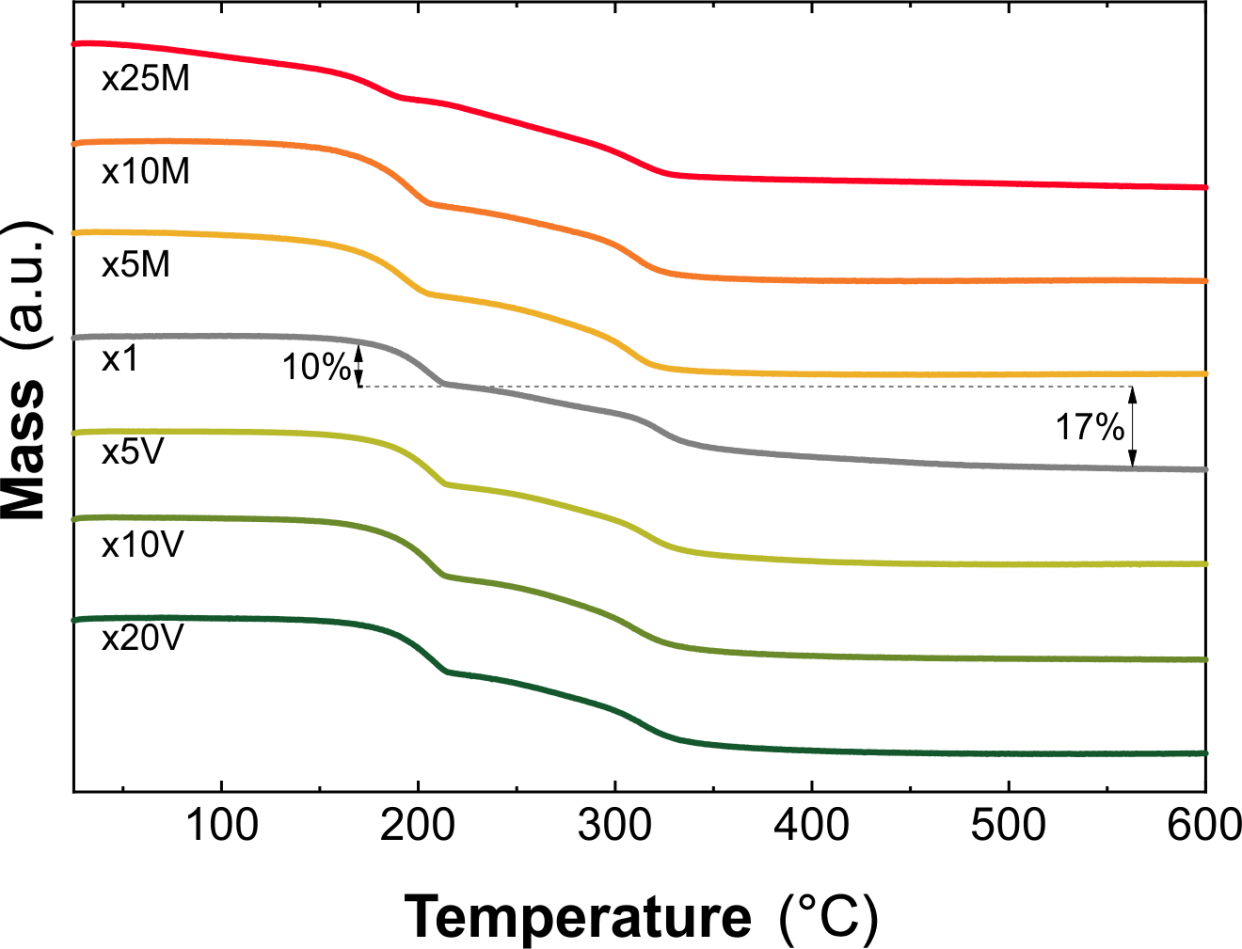
TGA analysis using a heating rate of 5 °C·min^−1^ in air of the scale-up samples.

Thermal decomposition in both inert (nitrogen) and oxidative (air) atmospheres was measured through thermogravimetric analysis (TGA). Typically, the decomposition of layered hydroxide structures consists of at least two main mass loss steps. The first, below 200 °C, is related to the release of physisorbed and interlayer water. The second one consists of the loss/decomposition of the interlayer anion and the concomitant dehydroxylation process, which leads to the collapse of the layered hydroxide structure [13,54]. The TGA curve of reference x1 in air (Figure 3) shows a first mass loss step of ca. 12% at 207 °C and a second one of around 14% at 276 °C, which are in agreement with the literature [13,15]. All pure scale-up samples exhibit the same TGA profile with only subtle differences in terms of mass loss percentage and decomposition temperature (see Supporting Information File 1, Table S4), which in principle could be related to morphological aspects, vide infra. As expected, the PXRD analysis of the calcined solids confirmed the formation of Co_2_AlO_4_ spinel (see Figure S10, Supporting Information File 1) [62]. Once again, sample x25M shows differences in terms of thermal behavior, resembling simonkolleite-like α-Co^II^ LH samples (see Figure S11, Supporting Information File 1) [30,52,55]. TGA curves in inert atmosphere can be found in Supporting Information File 1, Figure S12.

Inductively coupled plasma mass spectrometry (ICP-MS) was employed to assess quantitatively the precipitation of the cations. It confirmed a Co/Al ratio of 2.0 ± 0.1. Hence, the formation of pure CoAl LDH samples containing carbonate as interlayer anion and exhibiting the chemical formula 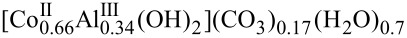 (see also Supporting Information File 1, Table S5 ) can be safely confirmed for both volumetric and mass scale-up approaches, up to 25-fold and tenfold, respectively.

Considering the experimental conditions for the production of pure CoAl-based LDH, we decided to compare the synthesis performance in terms of the space–time yield (STY). The STY value, defined as the amount of material (in kg) that can be produced per volume (in m^3^) per day, provides a good parameter to compare different synthesis protocols, as it has been demonstrated for metal-organic frameworks [63–65]. As expected, no-changes in STY values for the volumetric scale-up are observed, while in the case of the mass scale-up, there is a linear relation between STY values and initial concentration (Table 1). Supporting Information File 1, Table S6 compares the obtained STY values with those ones from other synthesis approaches such as co-precipitation and hydrothermal [39–41,65–69], mechanochemistry [27], and continuous flow methods [43–44].

**Table 1:** Space–time yield (STY) values for the synthesis procedures of pure CoAl-based LDH samples. In all cases, a synthesis time of 48 h is considered. Additionally, g·L^−1^ and L·kg^−1^ values are also provided.

Sample	STY (kg·m^3^·day^−1^)	g·L^−1^	L·kg^−1^

x1	0.23	0.46	2174

x5V	0.23	0.46	2174
x10V	0.23	0.46	2174
x20V	0.23	0.46	2174

x5M	1.15	2.3	434
x10M	2.3	4.6	217

**Figure 4:**
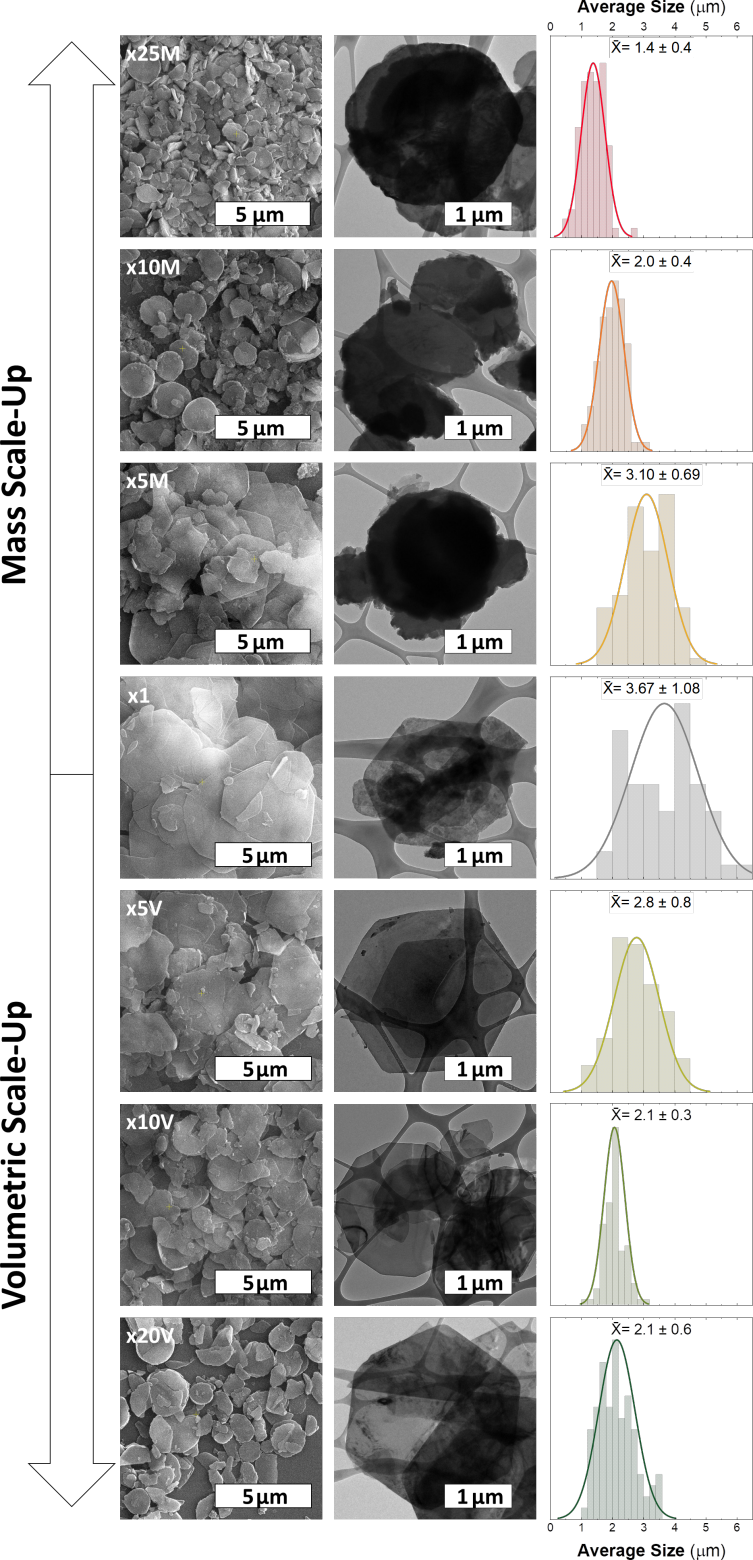
Microscopic characterization of CoAl-based LDH samples through (left) SEM and (center) TEM, and (right) the respective average size histograms.

**Figure 5:**
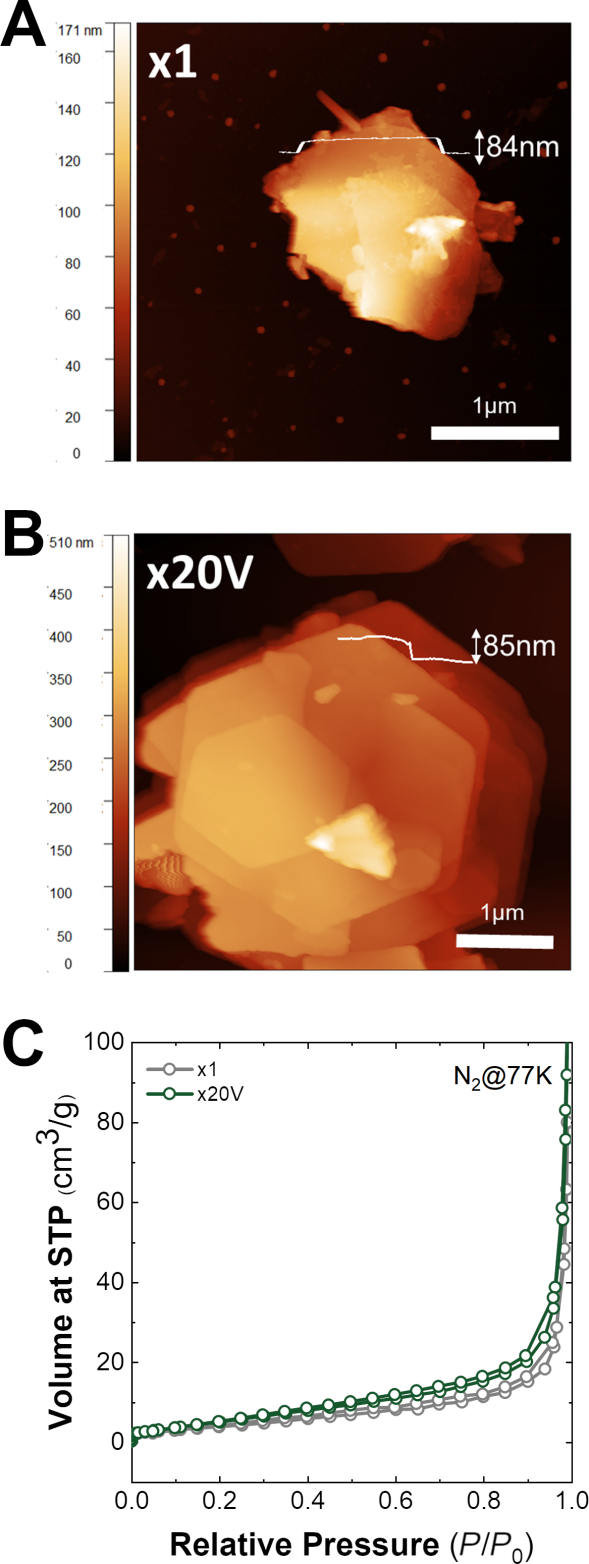
AFM images of the samples (A) x1 and (B) x20V. (C) Adsorption isotherms of samples x1 and x20V.

After the limits for the scale-up of CoAl-based LDH synthesis through an ARR method had been demonstrated, morphological aspects were addressed by means of scanning electron microscopy (SEM), transmission electron microscopy (TEM), and atomic force microscopy (AFM) (Figure 4 and Figure 5). For reference x1, well-defined hexagonal single crystals of around 3.7 ± 1.0 µm are observed, in good agreement with [13]. Interestingly, pure CoAl-based LDH scale-up samples exhibit a reduction of around 45% in size and a lack of sharp edges, regardless of the synthesis approach. In the case of mass scale-up protocols, these differences can arise from differences in the nucleation and growth processes because of increased concentration and ionic strength of the reagents, modifying the whole precipitation process [33]. However, the differences are surprising in the case of the volumetric scale-up approach where the initial concentrations were kept constant. Aiming to provide further information, the temperatures of the solutions were measured during the early stage of the volumetric experiments. According to Figure S13 (Supporting Information File 1), the required time to reach the final temperature increases sixfold from reference x1 to sample x20V, evidencing differences in heat transfer. Hence, considering that the precipitation kinetics is controlled by the alkalization process (i.e., the hydrolysis of urea, which depends on the temperature [35]), modifications in the heating process can affect the final size and shape of the particles [69]. This occurs surely through modification of the pristine Al-based hydroxide seeds [28,45,61,70], but even in processes where recrystallization can easily take place. Figure S14 and Table S7 (Supporting Information File 1) summarize the average size and standard deviation values as functions of the experimental conditions extracted from SEM analysis.

Besides slight differences in size and morphology (sharpness of the edges), the AFM comparison of single hexagonal platelets of reference x1 and sample x20V shows a similar thickness of around 85 nm (Figure 5A,B and Supporting Information File 1, Figure S15). Finally, textural properties were also evaluated by N_2_ and CO_2_ adsorption–desorption isotherms to observe possible changes in the surface area of the samples. Figure 5C shows the N_2_ isotherms at 77 K. The samples present type-IV adsorption isotherms (according to IUPAC classification) with an H3 hysteresis loop [71–76] and a low specific surface area (<50 m^2^/g) as previously reported [46]. Additional data, such as pore contributions (micro-, meso- and macropores) and other textural parameters, are compiled in Figure S16 and Table S8 (Supporting Information File 1).

To conclude, regarding the obtained pure CoAl-LDHs, both scale-up processes lead to subtle modifications of the morphological aspects, which can be understood in terms of changes in nucleation, growth and precipitation (mass scale-up), and different alkalization rates (volumetric approach).

## Conclusion

In this work, the effects of the scale-up of a CoAl-LDH synthesis have been examined for both mass and volumetric approaches. Pure CoAl-LDH can be obtained up to a tenfold concentration increase with subtle morphological modifications, related to changes in nucleation and growth (ionic strength increment). At a 25-fold concentration increase (x25M), the formation of a simonkolleite-like Co-based layered hydroxide impurity is observed, indicating phase competition during precipitation related to the thermodynamic stability of the growing phases. In the case of the volumetric scale-up, a reduction of ca. 45% of the particle size is observed as the volume increases. This feature is related to changes in the heating process (heat transfer) modifying the alkalinization kinetics and the concomitant precipitation process, even after 48 h of recrystallization. These results suggest that the final LDH morphology (size and sharpness and thickness of edges) is closely related to the growth of Al(OH)_3_-based seeds.

Hence, while the issues of the volumetric scale-up can be solved by accurate control of the heating process during the reaction, the drawbacks of the mass scale-up depend on the nature of the involved cations, requiring their specific optimization.

Overall, this work demonstrates, by means of several structural, microscopic, and spectroscopic techniques (including XANES-EXAFS synchrotron experiments), that the reproducible large-scale synthesis of high-quality morphologically controlled CoAl-LDHs is feasible, pinpointing the most critical synthesis aspects that should be controlled. Furthermore, this work offers reliable characterization fingerprints for controlling the quality and phase purity of these appealing anionic clays. These results may pave the way for the use of these 2D layered materials in different applications of great interest.

## Experimental

### Chemicals

Cobalt chloride hexahydrate (CoCl_2_·6H_2_O), aluminium chloride hexahydrate (AlCl_3_·6H_2_O), urea, and ethanol (EtOH) were purchased from Honeywell. All chemicals were used as received. Milli‐Q water was obtained from a Millipore Milli‐Q equipment.

### Synthesis

#### Synthesis method based on urea hydrolysis

The synthesis of the CoAl layered double hydroxide phase was carried out by hydrolysis of urea in a two-neck flask (with a reflux condenser) using 50 mL of an aqueous solution of the metal salts at 97 °C for 48 h under Ar atmosphere. The system was continuously stirred (750 rpm) during the whole synthesis process (heating, precipitation, and cooldown). Initial concentrations were fixed to [CoCl_2_] = 10 mM, [AlCl_3_] = 5 mM, and [urea] = 35 mM.

#### Volumetric scale-up

The volumetric scale-up of the LDH synthesis was carried out by using the same conditions as above, using two-neck round bottom flasks of 500, 1000, and 2000 mL with reaction volumes of 50, 250, 500, and 1000 mL, increasing the initial volume fivefold, tenfold, and 20-fold, respectively.

#### Mass scale-up

The mass scale-up of the LDH synthesis was carried out by using the same reaction volume as that of the urea hydrolysis, but increasing the concentration of reagents fivefold, tenfold, and 25-fold. The initial concentrations for each sample in the scale-up were fixed to (1) X5M: [CoCl_2_] = 50 mM, [AlCl_3_] = 25 mM, and [urea] = 175 mM; (2) X10M: [CoCl_2_] = 100 mM, [AlCl_3_] = 50 mM, and [urea] = 350 mM; and (3) X25M: [CoCl_2_] = 250 mM, [AlCl_3_] = 125 mM, and [urea] = 875 mM. All obtained solids were filtered, washed three times with H_2_O, H_2_O/EtOH, and finally with EtOH. The samples were dried at room temperature and kept in desiccators until further characterization.

#### Synthesis of α-Co layered hydroxide

The α-Co layered hydroxide synthesis was carried out at room temperature by using the epoxide route for a period of 48 h under constant stirring with solutions of [CoCl_2_] = 10 mM and [NaCl] = 100 mM, in the presence of glycidol, [Gly] = 500 mM.

### Chemical and structural characterization

Powder X-ray powder diffraction (PXRD) patterns were obtained on a PANalytical Empyrean X-ray platform with a capillary platform and copper radiation (Cu Kα = 1.54178 Å). Measurements were carried in triplicate in the 2-theta range of 2–70° with a step size of 0.02°/step and an integration time of 1 s.

Attenuated total reflectance Fourier-transform infrared spectroscopy (ATR-FTIR) spectra were collected on a Bruker alpha II FTIR spectrometer in the 4000–400 cm^−1^ range.

X-ray photoelectron spectroscopy (XPS) measurements were recorded on a Thermo Scientific™ K-alpha X-ray photoelectron spectrometer. Al Kα radiation was employed as X-ray source. For all elements, more than 100 spectra were recorded employing a step of 0.1 eV with a focused spot greater than 400 μm. XPS data were analyzed with the Thermo Avantage v5.9912 software. For the Co fits, FWHM values of 2.2, 2.9, 4.1, and 3.9 eV were employed for P_1_, P_2_, S_1_, and S_2_, respectively.

UV–vis absorption spectra of the solid samples were recorded in reflectance mode employing a Jasco V-670 spectrometer.

Thermogravimetric analysis (TGA) was carried out on a Netzsch TG 209 F1 Libra instrument in the 30–900 °C temperature range.

Magnetic data were collected over the bulk material with a Quantum Design superconducting quantum interference device (SQUID) MPMS-XL-5. The magnetic susceptibility of the samples was corrected considering the diamagnetic contributions of their atomic constituents as deduced from Pascal’s constant tables and the sample holder. The DC data were recorded under external applied fields of 100 or 1000 Oe in the 2–300 K temperature range. The AC data were collected under an applied field of 3.95 Oe at 997, 333, 110, 10, and 1 Hz. All magnetic measurements were carried out in eicosane, since this diamagnetic material allows for a better immobilization of these small anisotropic crystals, precluding any artefacts in the magnetic measurements.

The porous texture of all prepared materials was characterized by N_2_ adsorption at 77 K and CO_2_ at 273 K in an AUTOSORB-6 apparatus. Prior to the measurements, the samples were degassed for 4 h at 523 K and 5 × 10^−5^ bar. The desorption branch of the N_2_ isotherm was used to determine the pore size distribution using the BJH method. The surface area was determined using the BET method. The micropores volumes were determined by applying t-plot and DR methods.

### X-ray absorption spectroscopy

X-ray absorption spectroscopy (XAS) measurements were performed at the BL-22 (CLÆSS) beamline of the ALBA synchrotron (Barcelona, Spain), proposal: 2022097096. XANES and EXAFS Co K edge spectra were measured at room temperature in transmission mode. Absorbents of as-synthesized fresh samples were prepared by paint spraying on carbon paper. The optimum amount of material for the measurements was calculated by the program “Hephaestus”, which is part of the Demeter package [77]. A Si(111) double-crystal monochromator was used to obtain a monochromatic incident beam, and the intensities of incident and transmitted X-rays were measured using two ionization chambers. XAS spectra were collected from 7590 to 8550 eV with a reduced step (0.2 eV) in the XANES region (7690 to 7750 eV). The incident photon energy was calibrated using the first inflection point of the Co K edge (7709 eV) from a Co reference foil. For each sample, six spectra were taken with exposure times of 4 min each to later be averaged. XANES data treatment was performed by subtracting the pre-edge background followed by normalization by extrapolation of a quadratic polynomial fitted at the post-edge region of the spectrum using the ATHENA AUTOBK background removal algorithm [77]. The quantitative analysis of the EXAFS results were performed by modeling and ﬁtting the isolated EXAFS oscillations. The EXAFS oscillations χ(*k*) were extracted from the experimental data with standard procedures using the Athena program part of the Demeter package. The *k*^2^-weighted χ(*k*) data, to enhance the oscillations at higher *k*, were Fourier-transformed. The Fourier transformation was calculated using the sine filtering function. EXAFS modelling was carried out using the ARTEMIS software [77]. Theoretical scattering path amplitudes and phase shifts for all paths used in the fits were calculated using the FEFF9 code [78]. The *k* range was set from 2.3 to 12.1 Å^−1^. The passive reduction factor S_0_^2^ values were restrained to 0.8. This value was obtained from fitting a standard foil of metallic Co and constraining the coordination numbers to the corresponding structure.

### Microscopy

#### Sample preparation

The dried solids were suspended in ethanol and drop cast onto Au TEM grids covered with a lacy carbon film, and the solvent was left to evaporate. SEM samples were prepared from the same solution after 5 min of ultrasonication. The sonicated suspension was spin-coated on a Si wafer (3000 rpm, 40 s), washed with ethanol and dried afterward. For AFM, the samples were diluted in ethanol and drop-cast on a Si/SiO_2_ wafer. Si/SiO_2_ wafers were washed by spin-coating ten droplets of acetone and ten droplets of isopropanol prior to sample deposition.

#### Scanning electron microscopy (SEM)

Scanning electron microscopy data was acquired using a Hitachi S-4800, with a beam energy of 5 keV. The samples on silicon wafers were directly investigated without any surface coating. Energy dispersive X-ray (EDS) spectroscopy studies were performed on a Hitachi S-4800 microscope at an accelerating voltage of 20 kV.

#### Atomic force microscopy (AFM)

AFM was carried out with a Bruker Dimension Icon microscope in scan-assist-mode. A Bruker Scanasyst-Air silicon tip with a diameter of around 10 nm was used to obtain images with a resolution of 512 × 512 or 1024 × 1024 pixels. The Gwyddion software was used for flattening and image correction.

#### Transmission electron microscopy (TEM)

Transmission electron microscopy was carried out using a JEOL JEM-1010 at 100 kV accelerating voltage and a Tecnai F20 operated at 200 kV. Images were acquired in bright-field mode with an objective aperture selecting the unscattered electrons. To record the images, an AMT RX80 8MP CCD camera (JEOL JEM-1010) and a Gatan CCD 1k × 1k device were used.

## Supporting Information

Supporting Information features additional structural, spectroscopic, and magnetic characterization data.

File 1 Additional experimental data.

## References

[1] Geim A K, Novoselov K S (2007). Nat Mater.

[2] Miró P, Audiffred M, Heine T (2014). Chem Soc Rev.

[3] Mannix A J, Kiraly B, Hersam M C, Guisinger N P (2017). Nat Rev Chem.

[4] Carrasco J A, Congost-Escoin P, Assebban M, Abellán G (2023). Chem Soc Rev.

[5] Pastore H O, Marchese L (2009). J Mater Chem.

[6] Duong D L, Yun S J, Lee Y H (2017). ACS Nano.

[7] Liu Y, Weiss N O, Duan X, Cheng H-C, Huang Y, Duan X (2016). Nat Rev Mater.

[8] Coleman J N, Lotya M, O’Neill A, Bergin S D, King P J, Khan U, Young K, Gaucher A, De S, Smith R J (2011). Science.

[9] Centi G, Perathoner S (2008). Microporous Mesoporous Mater.

[10] Nicolosi V, Chhowalla M, Kanatzidis M G, Strano M S, Coleman J N (2013). Science.

[11] Kaul A B (2014). J Mater Res.

[12] Carrasco J A, Seijas‐Da Silva A, Oestreicher V, Romero J, Márkus B G, Simon F, Vieira B J C, Waerenborgh J C, Abellán G, Coronado E (2020). Chem – Eur J.

[13] Liu Z, Ma R, Osada M, Iyi N, Ebina Y, Takada K, Sasaki T (2006). J Am Chem Soc.

[14] Abellán G, Martí-Gastaldo C, Ribera A, Coronado E (2015). Acc Chem Res.

[15] SeijasDa Silva A, Sanchis-Gual R, Carrasco J A, Oestreicher V, Abellán G, Coronado E (2020). Batteries Supercaps.

[16] Yu J, Wang Q, O'Hare D, Sun L (2017). Chem Soc Rev.

[17] Chaillot D, Bennici S, Brendlé J (2021). Environ Sci Pollut Res.

[18] Mohapatra L, Parida K (2016). J Mater Chem A.

[19] Sarfraz M, Shakir I (2017). J Energy Storage.

[20] Fan G, Li F, Evans D G, Duan X (2014). Chem Soc Rev.

[21] Abellán G, Carrasco J A, Coronado E, Thomas S, Daniel S (2020). Layered Double Hydroxide Nanocomposites Based on Carbon Nanoforms. Layered Double Hydroxide Polymer Nanocomposites.

[22] Song X, Yuan F, Schoop L M (2021). Appl Phys Rev.

[23] Seijas-Da Silva A, Carrasco J A, Vieira B J C, Waerenborgh J C, Coronado E, Abellán G (2023). Dalton Trans.

[24] Mishra G, Dash B, Pandey S (2018). Appl Clay Sci.

[25] Miyata S (1980). Clays Clay Miner.

[26] Richetta M, Medaglia P G, Mattoccia A, Varone A, Pizzoferrato R (2017). J Mater Sci Eng.

[27] Qu J, Zhang Q, Li X, He X, Song S (2016). Appl Clay Sci.

[28] Oestreicher V, Jobbágy M (2013). Langmuir.

[29] He J, Wei M, Li B, Kang Y, Evans D G, Duan X, Duan X, Evans D G (2005). Preparation of Layered Double Hydroxides. Layered Double Hydroxides.

[30] Liu Z, Ma R, Osada M, Takada K, Sasaki T (2005). J Am Chem Soc.

[31] Ma R, Liu Z, Takada K, Fukuda K, Ebina Y, Bando Y, Sasaki T (2006). Inorg Chem.

[32] Abellán G, Coronado E, Martí-Gastaldo C, Ribera A, Jordá J L, García H (2014). Adv Mater (Weinheim, Ger).

[33] Okamoto K, Iyi N, Sasaki T (2007). Appl Clay Sci.

[34] Iyi N, Matsumoto T, Kaneko Y, Kitamura K (2004). Chem Lett.

[35] Arai Y, Ogawa M (2009). Appl Clay Sci.

[36] Xu Z P, Lu G Q (Max) (2005). Chem Mater.

[37] Ogawa M, Kaiho H (2002). Langmuir.

[38] Reichle W T (1986). Solid State Ionics.

[39] Sato T, Fujita H, Endo T, Shimada M, Tsunashima A (1988). React Solids.

[40] Kong X, Ge R, Liu T, Xu S, Hao P, Zhao X, Li Z, Lei X, Duan H (2021). Chem Eng J.

[41] Mao F, Hao P, Zhu Y, Kong X, Duan X (2022). Chin J Chem Eng.

[42] Chen Z, Fan Q, Huang M, Cölfen H (2022). CrystEngComm.

[43] Wang Q, Tang S V Y, Lester E, O'Hare D (2013). Nanoscale.

[44] Tichit D, Layrac G, Gérardin C (2019). Chem Eng J.

[45] Oestreicher V, Jobbágy M (2019). Chem – Eur J.

[46] Carrasco J A, Abellán G, Coronado E (2018). J Mater Chem C.

[47] Guoxiang P, Xinhui X, Jingshan L, Feng C, Zhihong Y, Hongjin F (2014). Appl Clay Sci.

[48] Sanchis-Gual R, Hunt D, Jaramillo C, Seijas-Da Silva Á, Mizrahi M, Marini C, Oestreicher V, Abellán G (2022). ChemRxiv.

[49] Biesinger M C, Payne B P, Grosvenor A P, Lau L W M, Gerson A R, Smart R S C (2011). Appl Surf Sci.

[50] Hunt D, Oestreicher V, Mizrahi M, Requejo F G, Jobbágy M (2020). Chem – Eur J.

[51] Neilson J R, Schwenzer B, Seshadri R, Morse D E (2009). Inorg Chem.

[52] Oestreicher V, Hunt D, Torres-Cavanillas R, Abellán G, Scherlis D A, Jobbágy M (2019). Inorg Chem.

[53] Oestreicher V, Hunt D, Dolle C, Borovik P, Jobbágy M, Abellán G, Coronado E (2021). Chem – Eur J.

[54] Oestreicher V, Abellán G, Coronado E (2020). Phys Status Solidi RRL.

[55] Oestreicher V, Dolle C, Hunt D, Fickert M, Abellán G (2022). Nano Mater Sci.

[56] Du Y, Ok K M, O'Hare D (2008). J Mater Chem.

[57] de A. A. Soler-Illia G J, Candal R J, Regazzoni A E, Blesa M A (1997). Chem Mater.

[58] de A. A. Soler-Illia G J, Jobbágy M, Regazzoni A E, Blesa M A (1999). Chem Mater.

[59] Intasa-Ard S (Grace), Ogawa M (2023). J Solid State Chem.

[60] Raimundo B, Kino D, Kitgawa N, Tokudome Y, Nunes C D (2023). Appl Clay Sci.

[61] Oestreicher V, Fábregas I, Jobbágy M (2014). J Phys Chem C.

[62] Masoud E M, El-Bellihi A-A, Bayoumy W A, Abdelazeem E S (2017). Ionics.

[63] Oestreicher V, Jobbágy M (2017). Chem Commun.

[64] Huo J, Brightwell M, El Hankari S, Garai A, Bradshaw D (2013). J Mater Chem A.

[65] Garzón-Tovar L, Carné-Sánchez A, Carbonell C, Imaz I, Maspoch D (2015). J Mater Chem A.

[66] Intasa-Ard S (Grace), Bureekaew S, Ogawa M (2019). J Ceram Soc Jpn.

[67] Intasa-ard S, Ogawa M (2022). Appl Clay Sci.

[68] Kino D, Tokudome Y, Vaz P D, Nunes C D, Takahashi M (2017). J Asian Ceram Soc.

[69] Iqbal M A, Fedel M (2019). Coatings.

[70] Boclair J W, Braterman P S (1999). Chem Mater.

[71] Tian Z, Li Q, Hou J, Pei L, Li Y, Ai S (2015). J Catal.

[72] Abellán G, Coronado E, Martí-Gastaldo C, Ribera A, Otero T F (2013). Part Part Syst Charact.

[73] Yang K, Yan L-g, Yang Y-m, Yu S-j, Shan R-r, Yu H-q, Zhu B-c, Du B (2014). Sep Purif Technol.

[74] Triantafyllidis K S, Peleka E N, Komvokis V G, Mavros P P (2010). J Colloid Interface Sci.

[75] Yun S K, Pinnavaia T J (1995). Chem Mater.

[76] Sing K S W (1985). Pure Appl Chem.

[77] Ravel B, Newville M (2005). J Synchrotron Radiat.

[78] Rehr J J, Kas J J, Vila F D, Prange M P, Jorissen K (2010). Phys Chem Chem Phys.

