# Supplementary material for: Upscaling the urea method synthesis of CoAl layered double hydroxides
Source: Beilstein J Nanotechnol. 2023 Sep 11;14:927–38. doi: 10.3762/bjnano.14.76 (PMC10509544; doi:10.3762/bjnano.14.76)
Supplement: File 1 — Additional experimental data. [file Beilstein_J_Nanotechnol-14-927-s001.pdf]

BEILSTEIN JOURNAL OF NANOTECHNOLOGY

Supporting Information

for

Upscaling the urea method synthesis of CoAl layered double hydroxides

Camilo Jaramillo-Hernández, Víctor Oestreicher, Martín Mizrahi and Gonzalo Abellán

Beilstein J. Nanotechnol. **2023,** *14,* 927–938. doi:10.3762/bjnano.14.76

Additional experimental data

License and Terms: This is a supporting information file under the terms of the Creative Commons Attribution License (https://creativecommons.org/licenses/by/4.0). Please note that the reuse, redistribution and reproduction in particular requires that the author(s) and source are credited and that individual graphics may be subject to special legal provisions.
The license is subject to the *Beilstein Journal of Nanotechnology* terms and conditions: (https://www.beilstein-journals.org/bjnano/terms)

Table S1: Crystallite size of the samples for both scale-up approaches.

**Sample**		***d*BS (Å)**	***a* (Å)**
x1		7.55	3.06
**Volumetric Scale-Up**	x5V	7.57	3.06
	x10V	7.53	3.08
	x20V	7.54	3.06
**Mass Scale-Up**	x5M	7.55	3.06
	x10M	7.58	3.08
	x25M	-	-

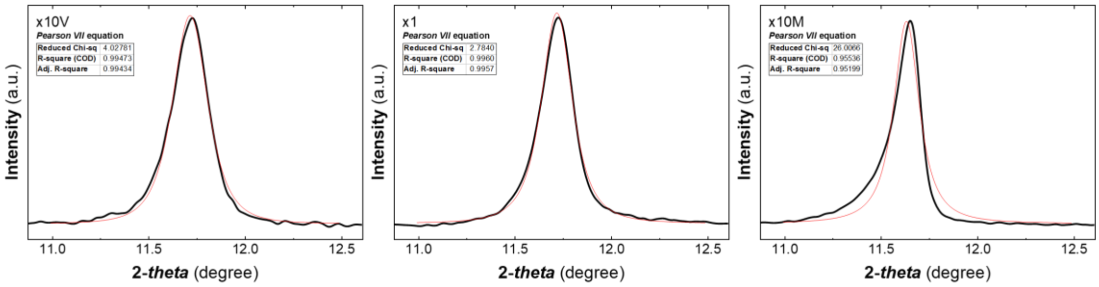

Figure S1: Main reflections (003) for samples x10V, x1, and x10M fitted by a Pearson VII function. A change in the symmetry of the peak is observed for the sample x10M, which could suggest the presence of an impurity.

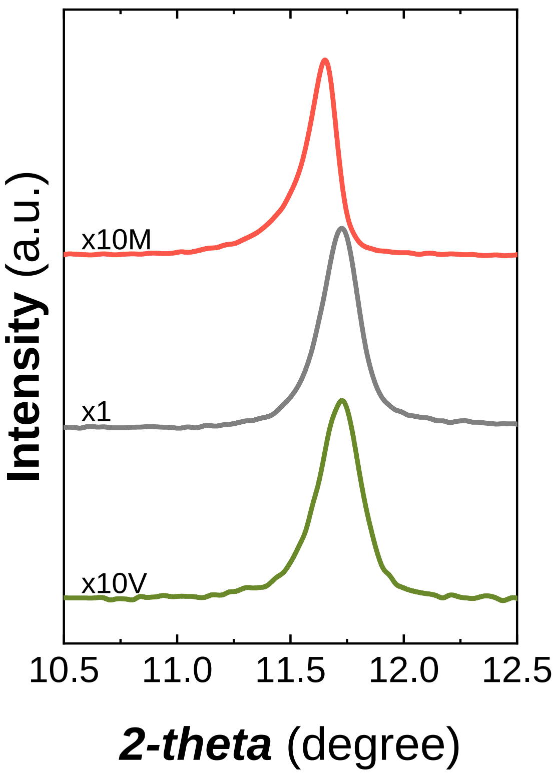

Figure S2: Detailed view of the (003) main reflection.

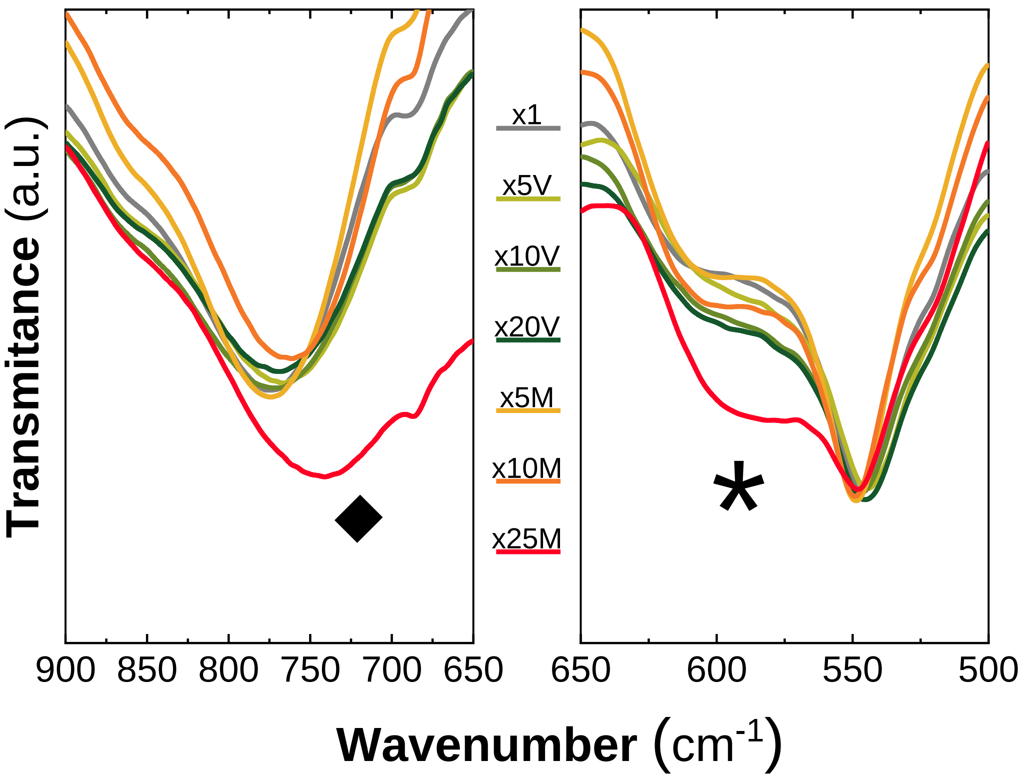

Figure S3: Normalized ATR-FTIR spectra depicting differences for the x25M sample. The diamond represents modifications in the carbonate signals (left), while the asterisk denotes a shoulder in the M-O band (right).

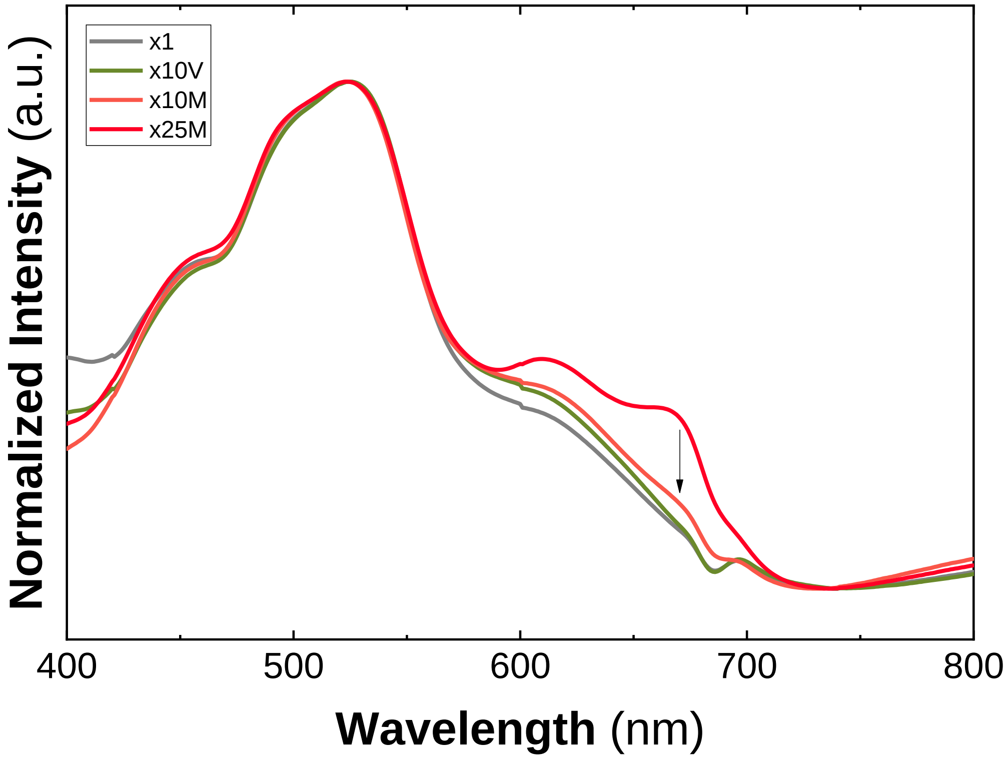

Figure S4: Normalized UV–vis spectra depicting differences for the samples x10 and x25M. A small peak at 670 nm for sample x10M is observed.

Table S2: XPS peaks summary.

	Co 2p $_{3/2}$				Co 2p $_{1/2}$			
	**P**		**S**		**P**		**S**	
	**1** (eV)	**2** (eV)	**1** (eV)	**2** (eV)	**1** (eV)	**2** (eV)	**1** (eV)	**2** (eV)
**X1**	781.3	783.2	786.9	790.8	797.4	798.6	803.5	806.5
**X20V**	781.2	783.1	785.9	789.5	797.4	799.2	803.1	805.7
**X25M**	781.2	783.1	786.3	789.7	797.3	798.7	803.0	805.4

	Al 2p $_{3/2}$			
	**P**		**S**	
	**1** (eV)	**2** (eV)	**1** (eV)	**2** (eV)
**X1**	74.2	74.7	76.5	76.8
**X20V**	74.1	74.4	75.7	76.0
**X25M**	74.2	74.7	76.4	76.8

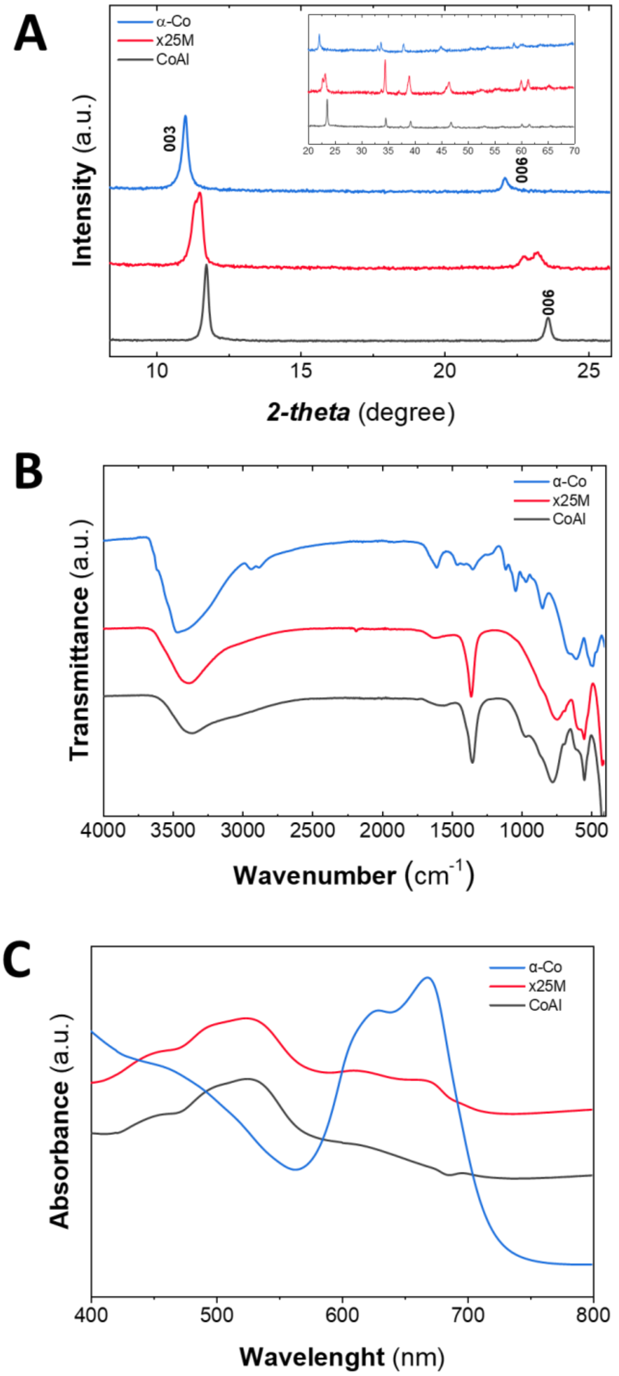

Figure S5: Comparison between the sample x25M, CoAl-based LDH, and α-Co LH by (A) PXRD, (B) ATR-FTIR spectroscopy, and (C) UV–vis spectroscopy.

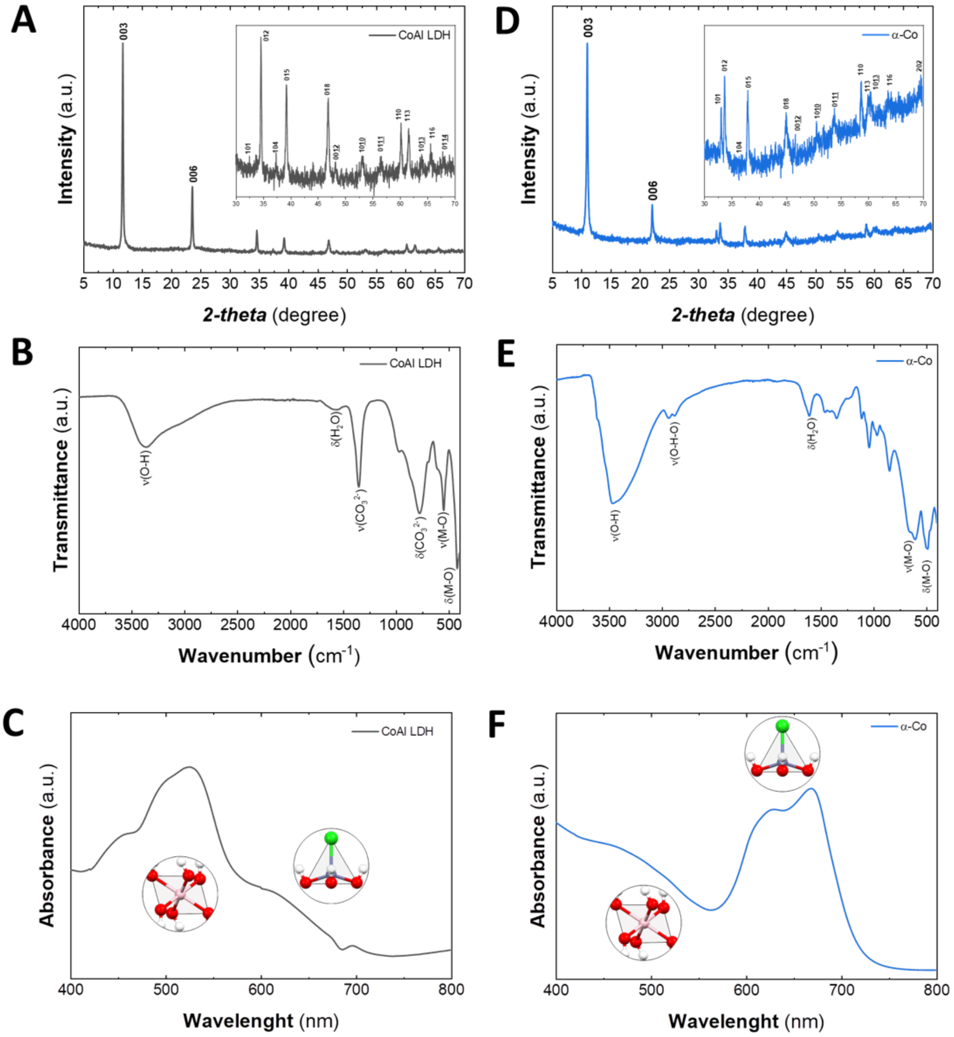

Figure S6: Comparative characterization of CoAl-based LDH (left) and simonkolleite-like α-Co-LH structures: (A, D) PXRD, (B, E) ATR-FTIR spectroscopy, and (C, F) UV–vis spectroscopy.

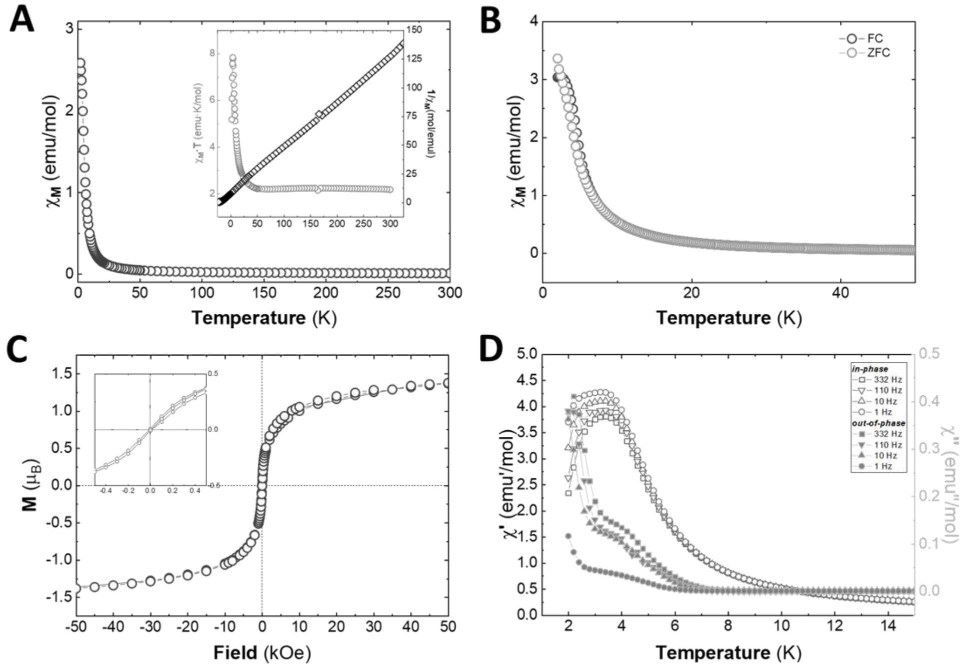

Figure S7: Magnetic characterization of the reference CoAl-LDH sample. (A) Magnetic susceptibility as a function of temperature with an external applied field of 1000 Oe; the inset represents the thermal dependence of $\chi_M \cdot T$ and the fit of $1/\chi_M$ to a Curie–Weiss law. (B) FC/ZFC with an external applied field of 100 Oe. (C) Hysteresis cycle at 2 K; the inset depicts the low-field region. (D) Thermal dependence of dynamic susceptibility for the in-phase (χ_M') and the out-of-phase (χ_M'') signals at 1, 10, 110, and 332 Hz.

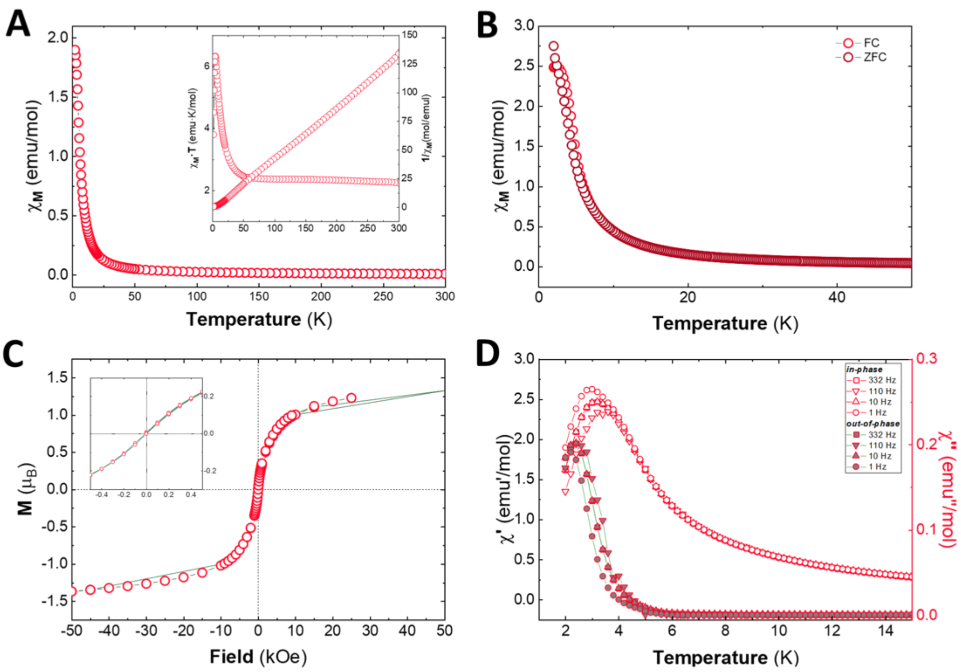

Figure S8: Magnetic characterization of the x25M CoAl-LDH sample (containing a simonkolleite-like Co-based LH impurity). (A) Magnetic susceptibility as a function of temperature with an external applied field of 1000 Oe; the inset represents the thermal dependence of $\chi_M \cdot T$ and the fit of the $1/\chi_M$ to a Curie–Weiss law. (B) FC/ZFC with an external applied field of 100 Oe. (C) Hysteresis cycle at 2 K; the inset depicts the low-field region. (D) Thermal dependence of dynamic susceptibility for the in-phase (χ_M') and the out-of-phase (χ_M'') signals at 1, 10, 110, and 332 Hz.

EXAFS analysis

Additionally, extended X-ray absorption fine structure region (EXAFS) measurements were carried out. Figure S9 depicts the magnitude of the Fourier transform (FT) of the EXAFS oscillations at the Co K edge with their corresponding fits. For all samples, the two major contributions, located in the range of 1–3 Å, represent the average distances (without phase correction) to the first and second coordination shell around the Co atoms (i.e., Co–O and Co–Co/Al, respectively). The samples x1 and x20V have a radial distribution with indistinguishable distances. In contrast, sample x25M exhibits a marked broadening of the first peak, in addition to an increase in the amplitude of the second peak, in comparison with pure x1 and x20V LDH samples. The structural parameters coordination numbers (*N*), interatomic distances (*R*), and structural disorder (σ^2) were obtained through EXAFS fits. In the case of samples x1 and x20V, the proposed model considers the following initial structure: a unique Co–O distance for O_h cations, plus a single shell of M (Co or Al), as second neighbors. However, for the x25M sample, it is necessary to add another Co–O coordination sphere with a smaller distance than the previous one (typically observed for $Co^{II}(T_d)$ moieties), while maintaining the M–M coordination shell invariant. In all cases, the high quality of the fits confirms a good match with the proposed models for each sample, reproducing the pseudo-radial distributions. Table S3 compiles the fit results. Thus, for the x1 and x20V samples, the distances to first and second neighbors are in good agreement with the crystallographic data and previous studies for a pure CoAl LDH structure [1]. Specifically, Co^{II}(Oh)–O = 2.1 Å and Co^{II}–Co^{II}/A^{III} = 3.1 Å, the average Co–O coordination number is ca. 6, while the Co–Co and Co–Al coordination numbers are close to 3.8 and 1.8, respectively, in both samples. Subtle differences in the Debye–Waller factor (σ^2) are found. It is higher in the x20V sample, which can be attributed to a greater structural disorder resulting from the differences in terms of size or morphology (vide infra). Finally, in the case of sample x25M, the two Co–O distances are associated to the presence of Co(O_h) and Co(T_d) environments, with the latter being attributed to the fraction of α-Co-LH structure. In the case of the second coordination sphere, similar distances are found corresponding to Co–Co (3.11 Å) and Co–Al (3.09 Å), the latter being slightly smaller for the three samples. The Co–Al coordination number decreases in the x25M sample, while the $N_{Co–Co}$ value increases. This is again a consequence of the presence of an α-LH phase fraction, which also explains the increase in the amplitude in the intensity of the second peak observed in Figure S9.

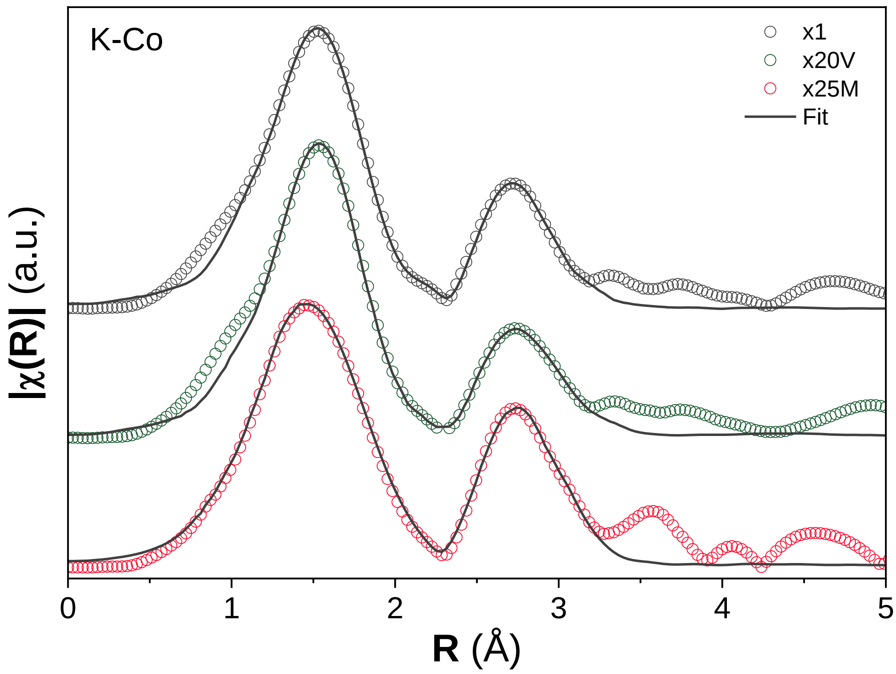

Figure S9: Fourier transform of the extracted EXAFS oscillations at the Co K edge for the measured samples (circles) and their corresponding fits (black line).

Table S3: Structural results from the EXAFS fit (*N*: average coordination number, *R*: average interatomic distance, and σ^2: Debye–Waller factor) at the Co K edge for the studied samples.

**Sample**	***1st shell***						***2nd shell***					
	1st environment $Co^{II}(T_d)$-O			***2nd environment $Co^{II}(O_h)$-O***			***1st environment Co^{II}- Co^{II}***			***2nd environment Co^{II} - Al^{II}***		
	N	***R*** (Å)	σ^2(Å^2)	***N***	***R*** (Å)	σ^2(Å^2)	***N***	***R*** (Å)	σ^2(Å^2)	***N***	***R*** (Å)	σ^2(Å^2)
x1				5.9	2.10	0.006	3.8	3.11	0.006	1.8	3.09	0.007
X20V				5.8	2.10	0.006	3.7	3.11	0.008	1.8	3.09	0.009
X25M	1.2	1.93	0.006	4.4	2.10	0.006	4.9	3.11	0.007	1.0	3.09	0.007

Table S4: Temperatures of decomposition and percent of mass loss per step of all samples.

Sample	Decomposition Temperature (ºC)		Mass loss (%)	
	1st step	2nd step	1st step	2nd step
**x1**	207	276	9.85	16.69
**x5V**	211	260	12.03	16.39
**x10V**	208	252	12.89	16.29
**x20V**	213	253	12.89	16.18
**x5M**	197	260	13.70	15.37
**x10M**	196	249	13.96	15.94
**x25M**	180	224	12.00	18.57

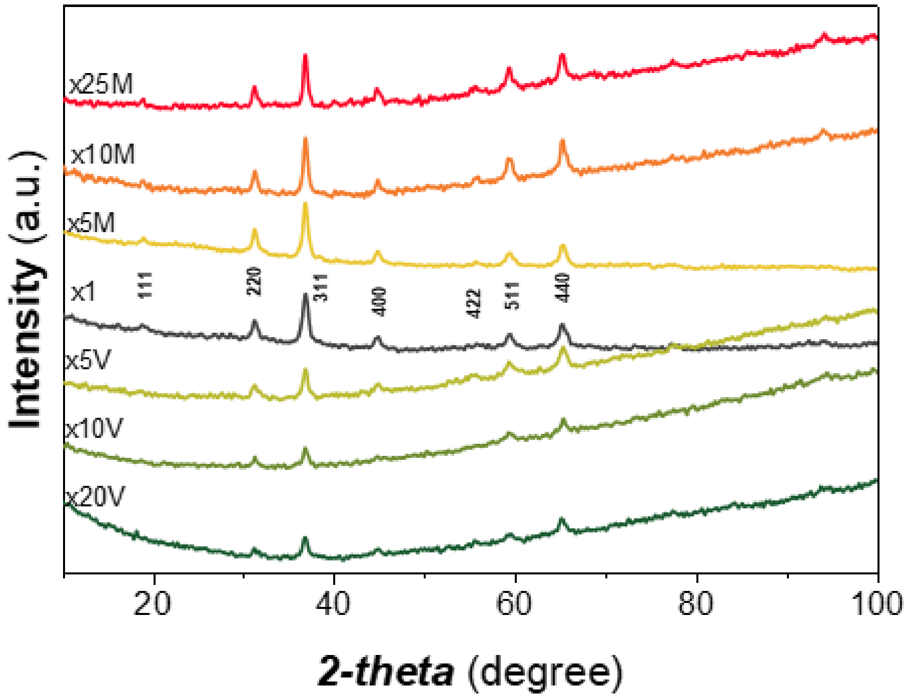

Figure S10: PXRD patterns obtained after TGA characterization in air (Figure 2, main manuscript).

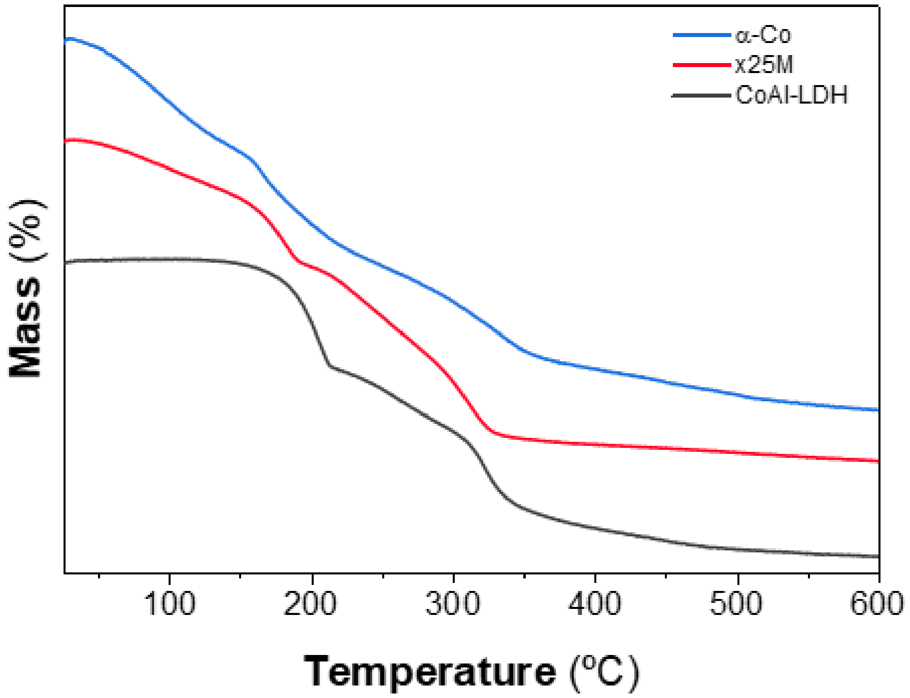

Figure S11: TGA comparison between α-Co LH, sample x25M and CoAl LDH.

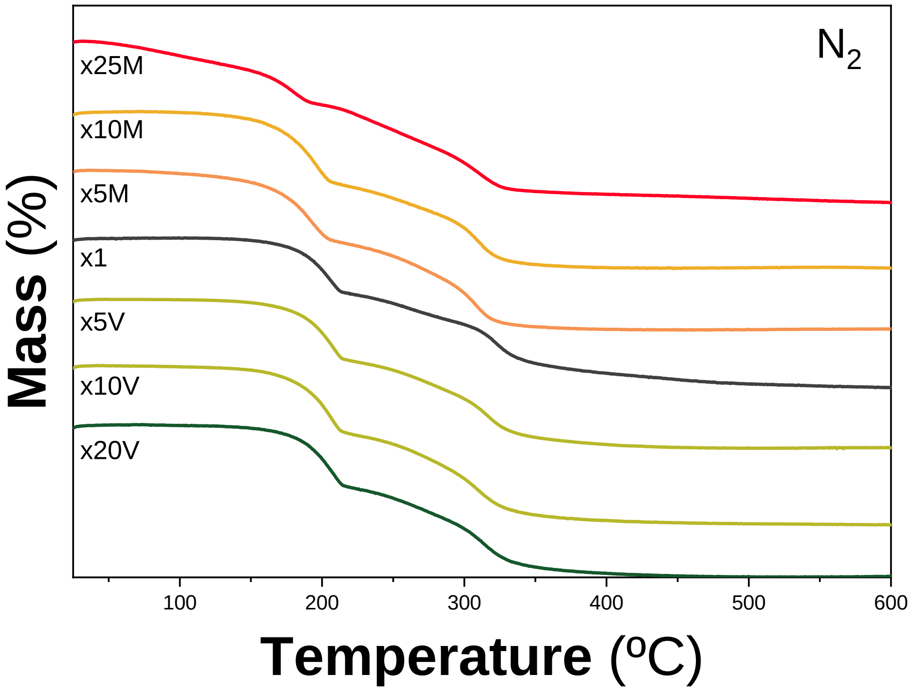

Figure S12: TGA using a heating rate of 5 °C·min^{-1} in an inert atmosphere for the scale-up samples.

Table S5: Amount of solvent (water) in all pure samples.

Sample	Water Molecules
**x1**	0.70
**x5V**	0.70
**x10V**	0.76
**x20V**	0.76
**x5M**	0.81
**x10M**	0.83
**X25M**	

Table S6: Yield values for different synthetic approaches, calculated with the information provided in their respective works.

LDH phases	Synthetic approach	STY *	Kg/day	L/kg	Ref
CoAl-based	Urea method	2.3	0.0023	217	Our work
CaAl-based	Co-precipitation	-	8220	-	*Chem. Eng. J.* **2021**, *407*, 127178.
CaAl-based	Co-precipitation	-	4110	-	*Chin. J. Chem. Eng.* **2022**, *41*, 42–48
CoAl-based	Co-precipitation	-	-	200	*React. Solids* **1988**, *5* (2–3), 219–228
CoAl-based	Co-precipitation	-	-	20	*Journal of Asian Ceramic Societies **2017** 5* 466–471
CoAl-based		-	-	13	*Applied Clay Science **2023*** 239 106948
MgAl-based	ARR Hydrothermal	-	-	19	*Journal of Solid State Chemistry **2023** 317* 123664
MgAl-based	Co-precipitation	-	-	14	*Applied Clay Science **2022** 228* 106615
MgAl-based	Co-precipitation			10	*Journal of the Ceramic Society of Japan **2019*** 127 11-17
MgAl-based	Mechanochemistry	-	-	300	*Appl. Clay Sci.* **2016**, *119*, 185–192
MgAl-based		-	-	240	
MgAlFe-based		-	-	225	
NiFe-based		-	-	400	
CoFe-based		-	-	400	
NiFe-based		-	-	300	
MgAl-based	Continuous flow	-	-	625	*Nanoscale* **2013**, *5* (1), 114–117
ZnAl-based	Continuous flow	-	-	300	*Chem. Eng. J.* **2019**, *369*, 302–332
MgAl-based		-	-	100	
NiFe-based		-	-	30	
MgAl-based		-	-	18	

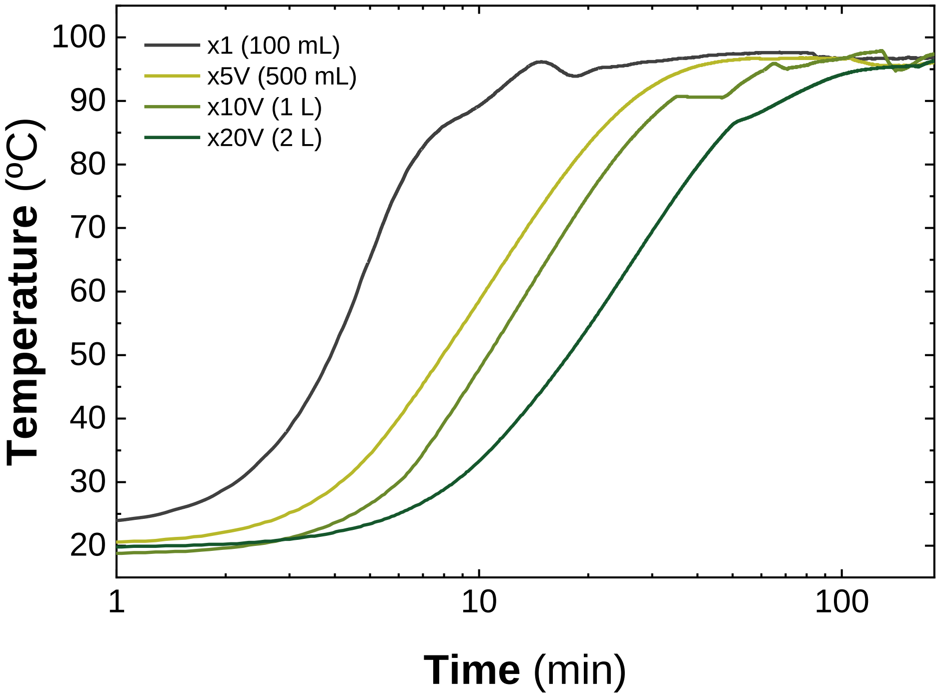

Figure S13: Temperature kinetics of the volumetric scale-up systems.

Table S7: Statistical size distribution (in µm) of all samples.

Sample	Average	SD	Min	Median	Max
**x1**	3.67	1.08	1.88	3.651	6.02
**x5V**	2.78	0.72	1.11	2.75	4.46
**x10V**	2.07	0.34	1.04	2.04	3.17
**x20V**	2.15	0.58	1.00	2.13	3.52
**x5M**	3.10	0.69	1.79	3.13	4.65
**x10M**	1.98	0.40	1.03	2.00	3.01
**x25M**	1.38	0.38	0.45	1.37	2.70

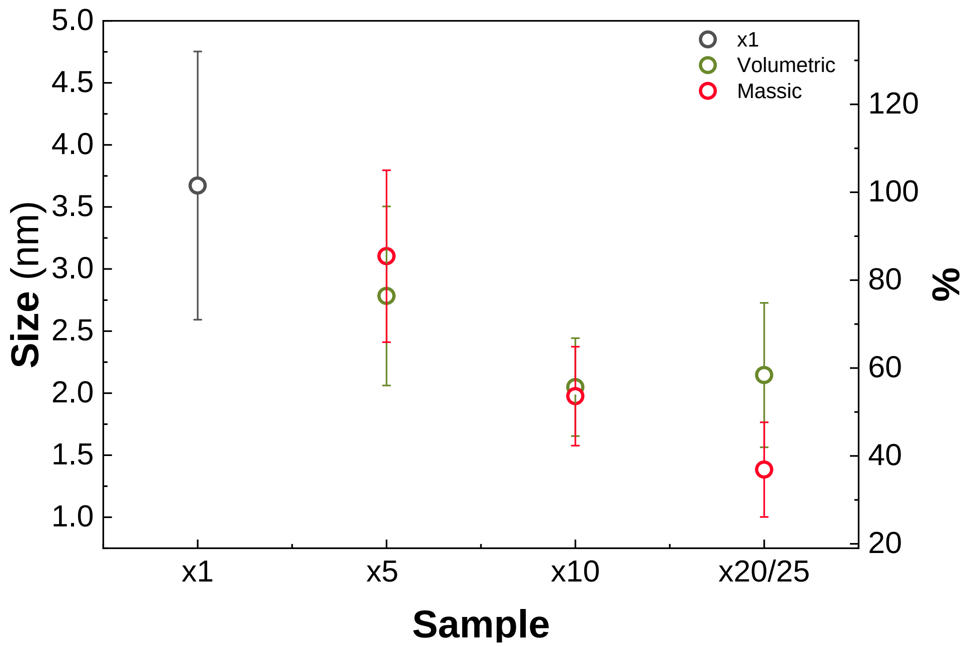

Figure S14: Size distribution from SEM analysis of the scale-up samples in comparison to reference x1.

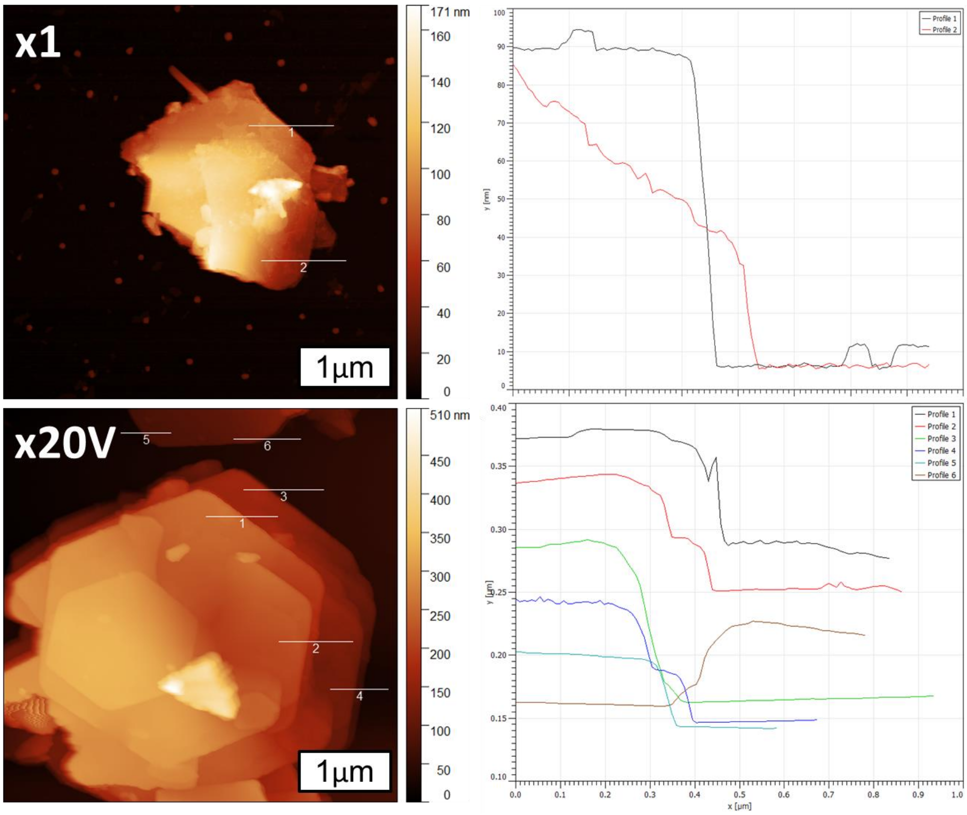

Figure S15: Thickness profiles of CoAl-LDH samples x1 and x20V.

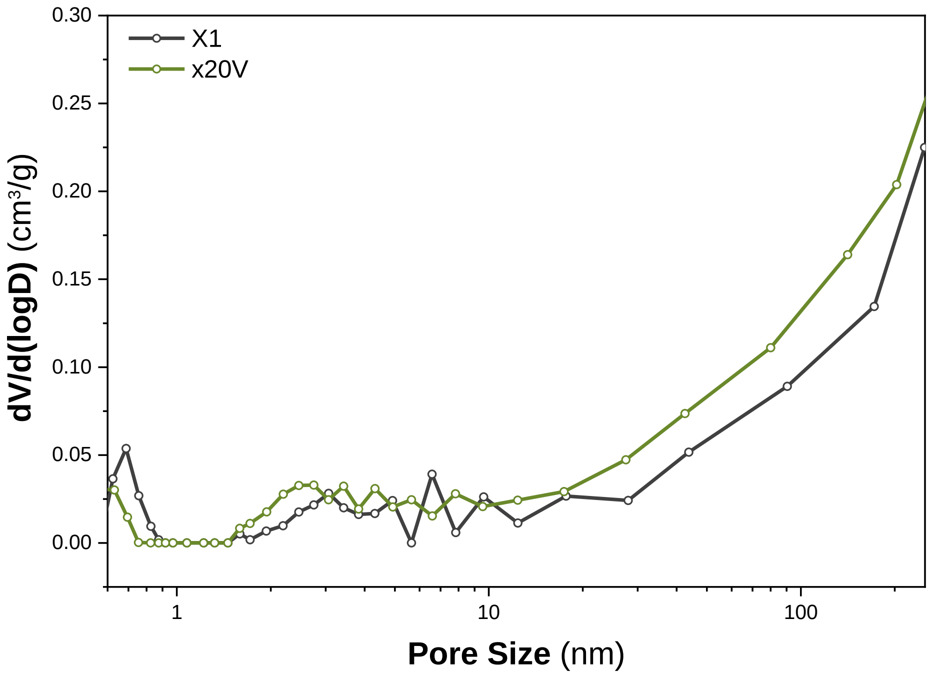

Figure S16: Pore distribution of CoAl-based LDH samples x1 and x20V.

Table S8: Textural parameters of CoAl-CO_3 LDH scale-up samples.

	S_{BET} a)	Tplot a)		V_{μ} (< 0.7 nm) b)	$V_{\mu DR}$ c)	$V_{\mu(0.7–2nm)}$ d)	V_{meso} e)
		S_{μ}	S_t				
	m^2/g	m^2/g	m^2/g	cm^3/g	cm^3/g	cm^3/g	cm^3/g
**X1**	13.264	0.000	13.264	0.042	0.004	0.038	0.002
**X20V**	21.748	0.000	21.748	0.021	0.004	0.017	0.002

[a)]Data obtained from N2 adsorption. Specific surface area calculated by BET method, and t-plot for the microporous surface contribution S_{μ} and external surface ST;
[b)]Data obtained from CO_2 adsorption. Micropore volume (<0.7 nm) calculated according to the Dubinin–Radushkevich (DR) method;
[c)]Micropore volume calculated from N_2 adsorption using DR method;
[d)]Micropore volume in the 0.7–2 nm range calculated according to: Vμ(0.7–2 nm) = VμDR − Vμ(<0.7 nm) values;
[e)]Mesopore volume was calculated according to: Vmeso =$V(P/P_0 = 0.7)$ − VμDR values.
